# Four New Phloroglucinol-Terpene Adducts from the Leaves of *Myrciaria cauliflora*

**DOI:** 10.1007/s13659-020-00288-4

**Published:** 2020-12-06

**Authors:** Ming Chen, Jia-Qing Cao, Wen-Jing Wang, Ni-Ping Li, Yan Wu, Lei Wang, Wen-Cai Ye

**Affiliations:** 1 Institute of Traditional Chinese Medicine & Natural Products, College of Pharmacy, Jinan University, Guangzhou, 510632 People’s Republic of China; 2 Guangdong Province Key Laboratory of Pharmacodynamic Constituents of TCM and New Drugs Research, Jinan University, Guangzhou, 510632 People’s Republic of China

**Keywords:** *Myrciaria cauliflora*, Phloroglucinol–terpene adducts, Antibacterial activity

## Abstract

**Abstract:**

Myrcauones A–D (**1**–**4**), four new phloroglucinol–terpene adducts were isolated from the leaves of *Myrciaria cauliflora*. Their structures with absolute configurations were elucidated by combination of spectroscopic analysis, single crystal X-ray diffraction, and electronic circular dichroism (ECD) calculations. Compound **1** was a rearranged isobutylphloroglucinol–pinene adduct featuring an unusual 2,3,4,4a,10,11-hexahydro-1*H*-3,11a-methanodibenzo[*b*,*f*]oxepin backbone. Compound **4** showed moderate antibacterial activity against Gram-positive bacteria including multiresistant strains.

**Graphic Abstract:**

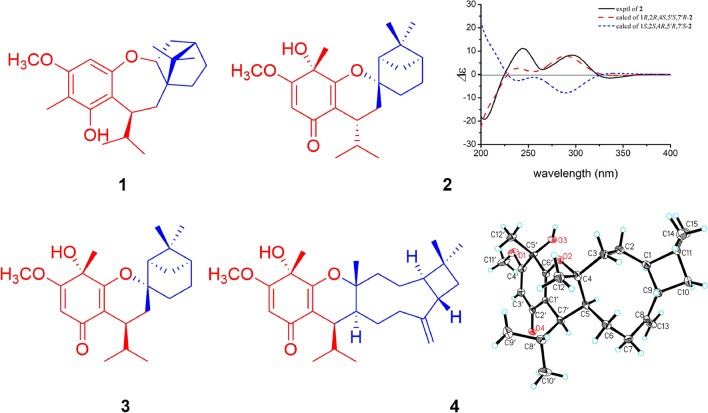

**Electronic supplementary material:**

The online version of this article (10.1007/s13659-020-00288-4) contains supplementary material, which is available to authorized users.

## Introduction

The plant *Myrciaria cauliflora* is an evergreen shrub and widely distributed in southern and central Brazil [1]. This plant has been traditionally used as a folk medicine to treat asthma, diarrhea, and gastrointestinal diseases [2, 3]. Previous phytochemical investigations on this plant only reported essential oils and flavonoids [4–6]. As a part of our efforts to search for structural unique and bioactive constituents from Myrtaceae plants [7–10], four new phloroglucinol–terpene adducts, myrcauones A–D (**1**–**4**), were isolated from the leaves of *M. cauliflora*. Their structures and absolute configurations were determined by means of 1D and 2D NMR spectroscopy, X-ray diffraction analysis, and electronic circular dichroism (ECD) calculations. Compound **1** is a rearranged isobutylphloroglucinol–pinene adduct featuring an unusual 2,3,4,4a,10,11-hexahydro-1*H*-3,11a-methanodibenzo[*b*,*f*]oxepin backbone. All isolates were evaluated for their antibacterial activities. Herein, we describe the isolation, structural elucidation, and antibacterial activities of these myrcauones A–D (**1**–**4**).

## Results and Discussion

### Structural Elucidation

**Fig. 1:**
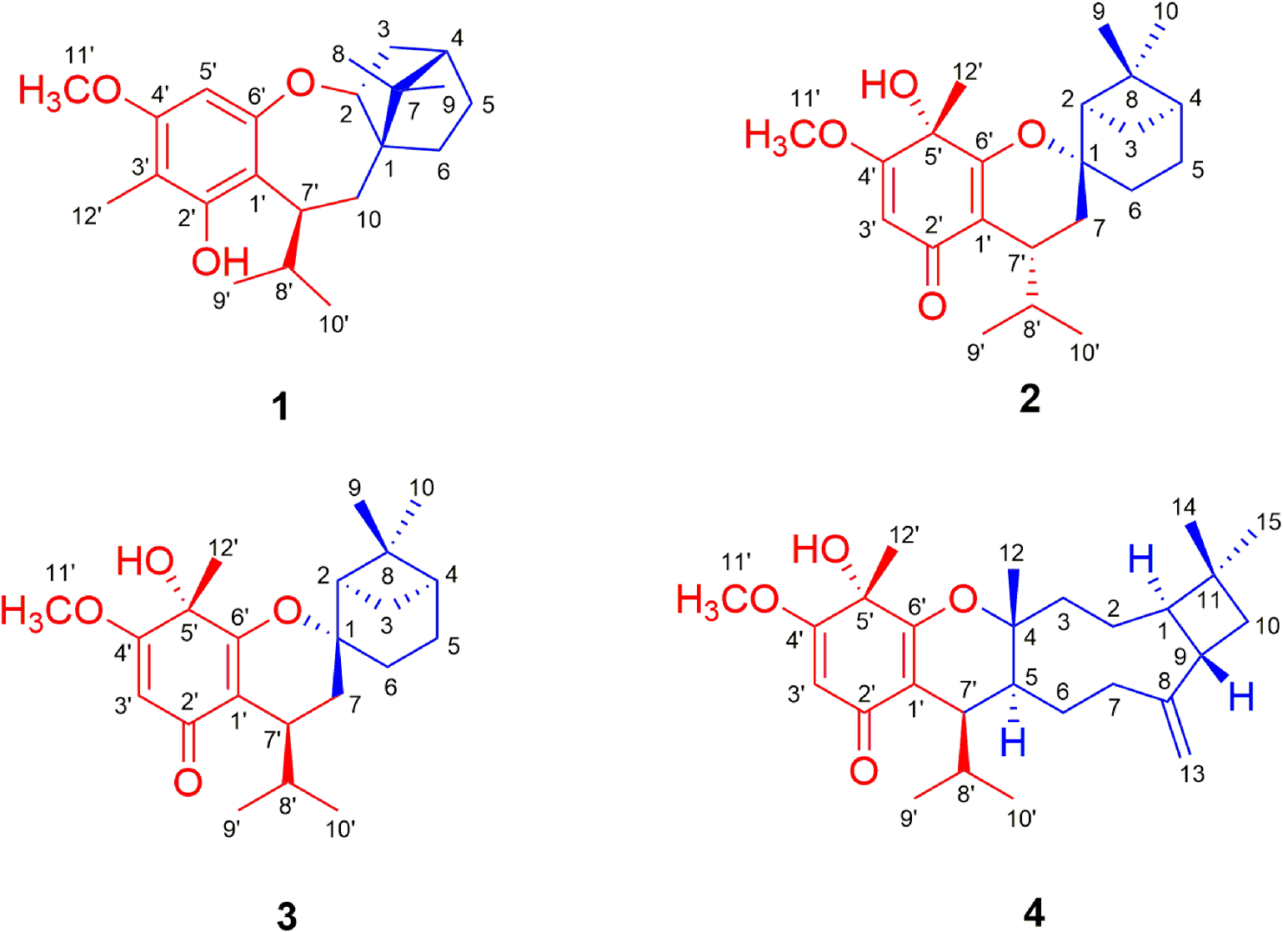
Chemical structures of **1**–**4**

Compound **1** was obtained as yellow gum. The molecular formula of **1** was established as C_22_H_32_O_3_ by its HRESIMS data (*m*/*z* 345.2423 [M+H]^+^, calcd for C_22_H_33_O_3_: 345.2424). The UV spectrum displayed absorption maximum at 206 nm. The IR spectrum showed characteristic absorptions for hydroxyl group (3475 cm^−1^) and aromatic ring (1611 and 1488 cm^−1^). The ^1^H NMR spectrum of **1** suggested the presence of an olefinic proton [*δ*_H_ 6.23 (1H, s, H-5′)], a hydroxyl group [*δ*_H_ 4.75 (1H, s, 2′-OH)], a methoxy group [*δ*_H_ 3.76 (3H, s, H_3_-11′)], an isopropyl moiety [*δ*_H_ 1.97 (1H, m, H-8′), 0.52 (3H, d, *J* = 6.8 Hz, H_3_-9′), and 1.03 (3H, d, *J* = 6.8 Hz, H_3_-10′)], and three tertiary methyls [*δ*_H_ 2.06 (3H, s, H_3_-12′), 0.90 (3H, s, H_3_-9), and 0.79 (3H, s, H_3_-8)]. The ^13^C NMR and DEPT spectra of **1** exhibited 22 carbon signals including 7 quaternary carbons (5 olefinic ones), 5 methines (an oxygenated and an olefinic ones), 4 methylenes, and 6 methyls (an oxygenated one). The aforementioned data implied that **1** could be an isobutylphloroglucinol–monoterpene adduct (Fig. 1) [11].

**Fig. 2:**
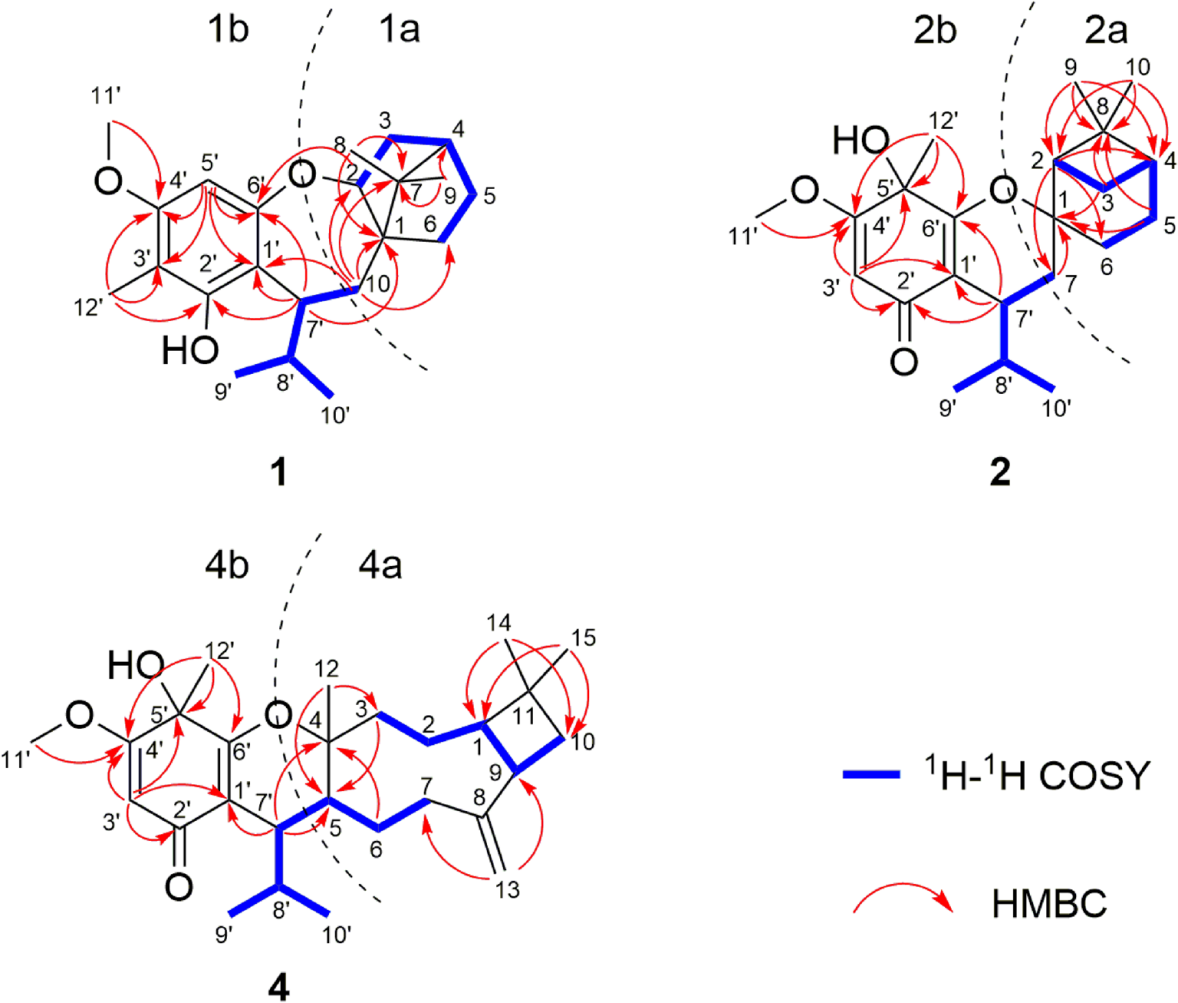
Key ^1^H–^1^H COSY and HMBC correlations of **1**, **2**, **4**

A comparison of the NMR data of **1** with those of melaleucadine A [11] indicated the presence of an uncommon rearranged *β*-pinene unit (part **1a**), which was further confirmed by the two spin systems (H-2 to H-6 and H-10 to H-9′/H-10′) in its ^1^H–^1^H COSY spectrum (Fig. 2) and the HMBC correlations between H_3_-8/H_3_-9 and C-1/C-4/C-7 and between H_2_-10 and C-1/C-2/C-6/C-7. In addition, the HMBC correlations between H-5′ and C-1′/C-3′/C-4′/C-6′, between H_3_-12′ and C-2′/C-3′/C-4′, between H_3_-11′ and C-4′, and between H-7′ and C-1′/C-2′/C-6′, allowed the establishment of an isobutylphloroglucinol moiety (part **1b**). Furthermore, the HMBC correlations between H-7′ and C-1, and between H_2_-10 and C-1′ defined the connection of **1a** and **1b** via C-7′–C-10 bond. Finally, the leftover oxygen atom was anticipated to connect C-2 (*δ*_C_ 88.6) with C-6′ (*δ*_C_ 159.4) to form an uncommon 2,3,4,5-tetrahydrooxepine ring on the basis of the HMBC correlation between H-2 and C-6′ as well as the molecular formula information.

**Fig. 3:**
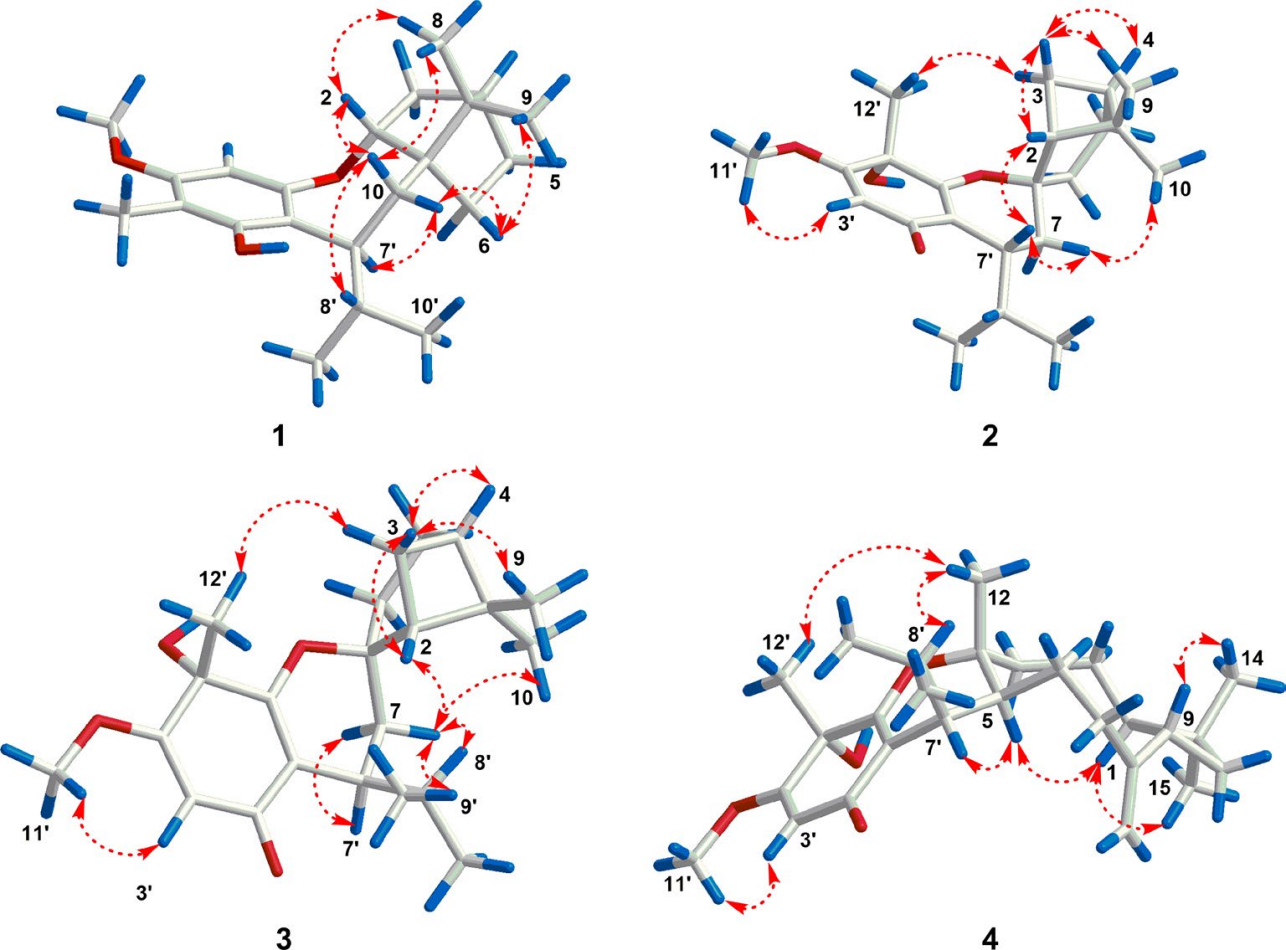
Key NOESY correlations of **1**–**4**

**Fig. 4:**
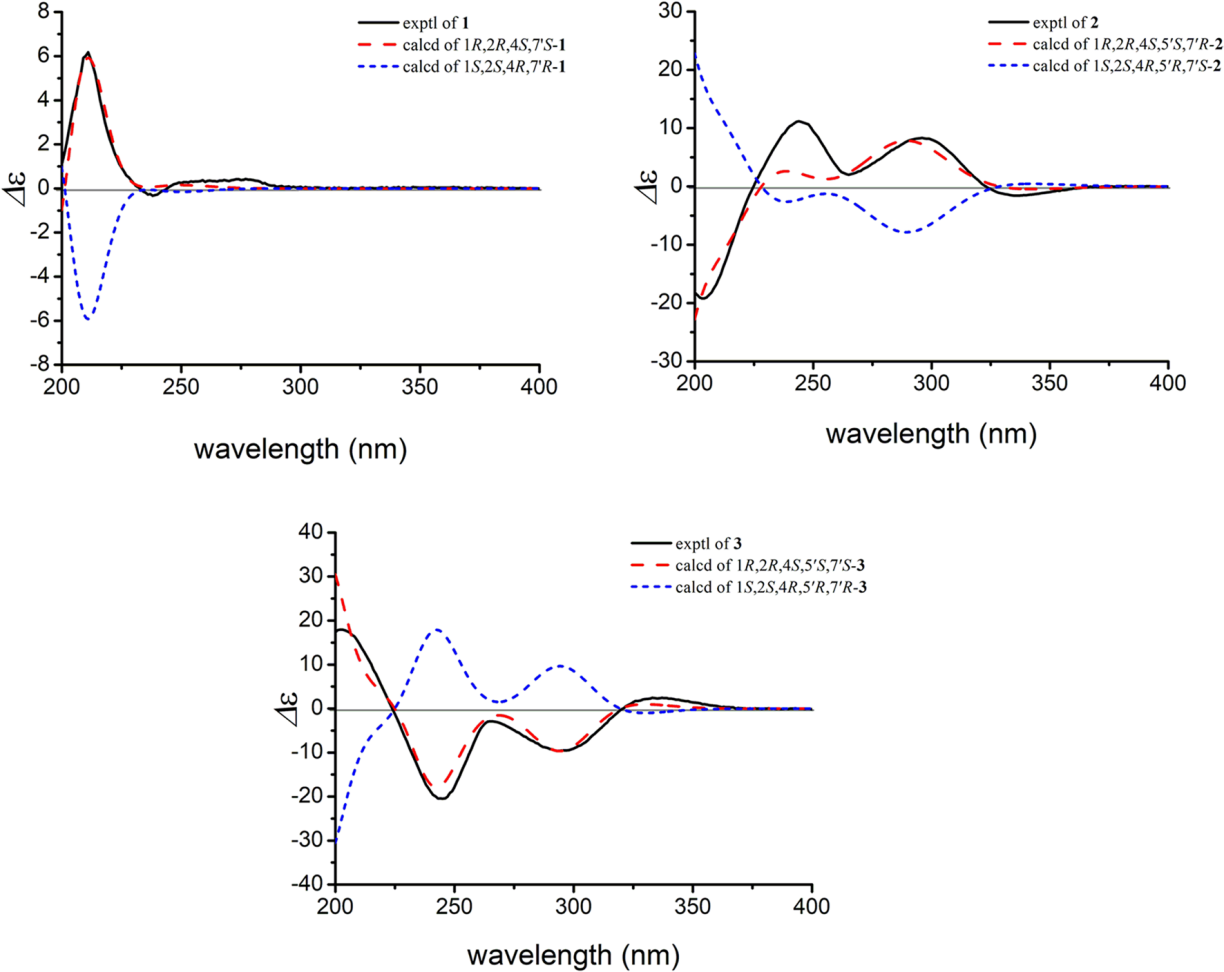
Calculated and experimental ECD spectra of **1**–**3**

The relative configuration of **1** was established by a NOESY experiment (Fig. 3). The NOE correlations between H-10*β* and H-2/Me-8/H-8′ indicated that H-2, Me-8, and the isopropyl group (C-9′/8′/10′) were *β*-oriented. Meanwhile, the correlations between H-10*α* and H-6a/H-7′ as well as between H-6a and Me-9 suggested that Me-9 and H-7′ were *α*-oriented. To determine the absolute configurations of **1**, a comparison of its experimental and calculated ECD data was performed. The experimental ECD spectrum of **1** exhibited negative Cotton effects at 211 (Δ*ε* + 6.2) and 277 (Δ*ε* + 0.7) nm, and a negative one at 238 (Δ*ε* − 0.5) nm, which were similar with those in the calculated CD spectrum for 1*R*,2*R*,4*S*,7′*S*-isomer (Fig. 4). Thus, the absolute configuration of **1** was determined as 1*R*, 2*R*, 4*S*, and 7′*S*.

The molecular formula of compound **2** was determined as C_22_H_32_O_4_ by its HRESIMS data (*m*/*z* 361.2386 [M+H]^+^, calcd for C_22_H_33_O_4_: 361.2373). The IR spectrum showed absorptions of hydroxyl (3357 cm^−1^), carbonyl group (1656 cm^−1^), and double bonds (1605 and 1462 cm^−1^). The ^1^H NMR data (Table 1) for an olefinic proton [*δ*_H_ 5.33 (1H, s, H-3′)], a methoxy group [*δ*_H_ 3.75 (3H, s, H_3_-11′)], an isopropyl moiety [*δ*_H_ 2.86 (1H, m, H-8′), 0.61 (3H, d, *J* = 6.8 Hz, H_3_-9′), and 0.93 (3H, d, *J* = 6.8 Hz, H_3_-10′)], and three tertiary methyls [*δ*_H_ 1.51 (3H, s, H_3_-12′), *δ*_H_ 1.23 (3H, s, H_3_-9), and 0.96 (3H, s, H_3_-10)] indicated that **2** could be an isobutylphloroglucinol–monoterpene adduct [12].

**Table 1:** ^1^H and ^13^C NMR data of **1–4** in CDCl_3_ (*δ* in ppm, *J* in Hz)

Nos.	**1**^a^		**2**^b^		**3**^b^		**4**^a^	
	*δ*_H_	*δ*_C_	*δ*_H_	*δ*_C_	*δ*_H_	*δ*_C_	*δ*_H_	*δ*_C_
1	–	52.5	–	85.4	–	84.7	1.53	58.1
2	3.85 (ddd 10.8, 4.8, 2.4)	88.6	2.22 (t, 5.2)	45.2	2.10 (t, 5.2)	53.0	1.56	23.6
3	*α* 1.21 (dd 13.6, 4.4)	35.7	a 1.53	26.4	a 1.67 (brd, 10.2)	26.5	*α* 1.48	46.0
	*β* 2.28 m		b 2.12		b 2.33		*β* 2.06	
4	1.62	44.3	1.96	40.8	1.98	40.6	–	85.8
5	a 1.34 (td 10.6, 3.2)	29.1	a 1.99	24.7	1.87	25.2	1.74	39.9
	b 1.76 m		b 1.89					
6	a 2.39 m	25.6	a 2.23	30.8	a 2.33	27.4	*α* 1.74	23.9
	b 1.64		b 1.89		b 1.87		*β* 1.69	
7	-	49.0	*α* 1.66 (dd, 14.0, 12.4)	32.7	*α* 1.90 (dd, 13.6, 6.8)	33.7	*α* 2.36	35.6
			*β* 2.07 (dd, 14.0, 6.4)		*β* 1.38 (dd, 13.6, 12.0)		*β* 2.18	
8	0.79 (s)	19.0	–	38.4	–	38.4	–	151.1
9	0.90 (s)	20.5	1.23 (s)	27.7	1.31 (s)	27.7	2.43	41.3
10	*α* 1.94 (dd 14.4, 3.6)	28.1	0.96 (s)	23.2	0.99 (s)	23.6	*α* 1.71 (t 10.4)	36.5
	*β* 1.44 (dd 14.4, 4.4)						*β* 1.57	
11							–	34.7
12							1.36 (s)	22.8
13							a 4.88 (s)	111.2
							b 4.90 (s)	
14							0.96 (s)	21.8
15							0.93 (s)	29.8
1′	–	115.9	–	111.1	–	112.4	–	113.0
2′	–	153.7	–	186.7	–	186.6	–	186.2
3′	–	105.4	5.33 (s)	100.0	5.35 (s)	100.2	5.37 (s)	99.7
4′	–	156.0	–	170.9	–	170.8	–	170.7
5′	6.23 (s)	97.2	–	70.0	–	69.8	–	69.3
6′	–	159.4	–	164.9	–	163.9	–	164.3
7′	3.09 (dt 11.2, 4.0)	42.3	2.59 (ddd, 12.0, 6.4, 4.4)	33.6	2.73 (ddd, 11.2, 6.8, 4.0)	32.2	2.66 dd (4.0, 3.2)	34.1
8′	1.97 (m)	32.0	2.86 (m)	26.3	2.92 (m)	26.2	2.07 (m)	26.0
9′	0.52 d (6.8)	21.8	0.61 (d, 6.8)	15.8	0.59 (d, 6.8)	15.6	0.65 d (6.8)	19.9
10′	1.03 (d 6.8)	22.8	0.93 (d, 6.8)	20.7	0.92 (d, 6.8)	20.6	1.13 d (6.8)	26.4
11′	3.76 (s)	55.6	3.75 (s)	56.2	3.76 (s)	56.2	3.76 (s)	56.2
12′	2.06 (s)	8.4	1.51 (s)	27.3	1.57 (s)	26.7	1.60 (s)	26.8
2′-OH	4.75 (s)							

Overlapped signals were reported without designating multiplicity

^a^Recorded at 500 (^1^H) and 125 (^13^C) MHz

^b^Recorded at 400 (^1^H) and 100 (^13^C) MHz

The ^1^H–^1^H COSY spectrum of **2** revealed the presence of two spin systems (H-2 to H-6 and H-7 to H-9′/H-10′) (Fig. 2). Accordingly, a *β*-pinene unit (part **2a**) could be established by the HMBC correlations between H-2 and C-4/C-6/C-7, between H_2_-3/H_2_-5 and C-1/C-8, and between H_3_-9/H_3_-10 and C-2/C-4/C-8. Furthermore, comparison of its NMR data with those of the known compound baeckfrutone H indicated the existence of an isobutyrylphloroglucinol moiety (part **2b**), which was further confirmed by the HMBC correlations between H-3′ and C-1′/C-2′/C-4′/C-5′, between H_3_-12′ and C-4′/C-5′/C-6′, between H_3_-11′ and C-4′, and between H-7′ and C-1′/C-2′/C-6′ [12]. The closure mode of dihydropyran ring which connected the two fragments (**2a** and **2b**) could be deduced on the basis of the molecular formula information and the downfield chemical shift at C-1 (*δ*_C_ 85.4).

In the NOESY spectrum, the correlations between H-3b and H-2/H-4/Me-9, between H-2 and H-7′, between H-7*β* and Me-10/H-7′, as well as between H-3a and Me-12′ indicated that these protons were all *β*-oriented (Fig. 3). The absolute configuration of **2** was determined by ECD calculation. The experimental ECD spectrum of **2** displayed positive cotton effects at 244 (+ 11.7) and 296 (+ 8.1) nm, and negative ones at 203 (− 19.8) and 337 (− 1.8) nm, which were similar to those in the calculated spectrum for 1*R*,2*R*,4*S*,5′*S*,7′*R*-**2** (Fig. 4). Thus, the absolute configuration of **2** was identified as 1*R*, 2*R*, 4*S*, 5′*S*, 7′*R*.

Compound **3** possessed the same molecular formula as **2** on the basis of its HRESIMS data (*m*/*z* 361.2391 [M+H]^+^, calcd for C_22_H_33_O_4_, 361.2373). Analyses of the NMR data of **3** and comparison with those of **2** indicated that these two compounds had the same planar structure but differed in their relative configurations. The downfield chemical shifts of C-2 (from *δ*_C_ 45.2 to 53.0) and C-7 (from *δ*_C_ 32.7 to 33.7), as well as the upfield chemical shifts of C-6 (from *δ*_C_ 30.8 to 27.4), C-7′ (from *δ*_C_ 33.6 to 32.2) revealed that **3** was a C-7′ epimer of **2**. This deduction was confirmed by the NOE correlations between H-3b and H-2/H-4/Me-9, between H-2 and H-7*β*, between H-7*β* and H-8′/Me-9′/Me-10, between H-3a and Me-12′, and between H-7*α* and H-7′ (Fig. 3). Finally, the agreement of the ECD curve of **3** with those of the calculated 1*R*,2*R*,4*S*,5′*S*,7′*S*-**3** (Fig. 4) allowed the assignment its absolute configuration.

Compound **4** was obtained as colorless blocks. Its molecular formula was determined to be C_27_H_40_O_4_ by its HRESIMS data at *m*/*z* 429.2996 [M+H]^+^ (calcd for C_27_H_41_O_4_: 429.2999). Comparison of the NMR data of **4** with those of **3** suggested that they had the same isobutyrylphloroglucinol moiety (part **4b**) (Fig. 2). The remaining NMR signals for 15 carbons implied the presence of a sesquiterpene moiety. The spin systems (from H-3 to H-10 and from H-7 to H-9′/H-10′) established by the ^1^H–^1^H COSY spectrum as well as the HMBC correlations between H_3_-14/ H_3_-15 and C-1/C-10, between H_2_-13 and C-7/C-9, and between H_3_-12 and C-3/C-5 indicated the presence of a caryophyllene unit (part **4a**) (Fig. 2), which was further confirmed by comparison of the NMR data of **4** with those of myrtucommulone K [10]. Furthermore, the HMBC correlations between H-7′ and C-4/C-5/C-1′ indicated the connection of parts **4a** and **4b** via a C-5 and C-7′ bond. Finally, the closure mode of dihydropyran ring which connected the two fragments (**4a** and **4b**) could be deduced on the basis of the molecular formula information and the downfield chemical shift at C-4 (*δ*_C_ 85.8).

**Fig. 5:**
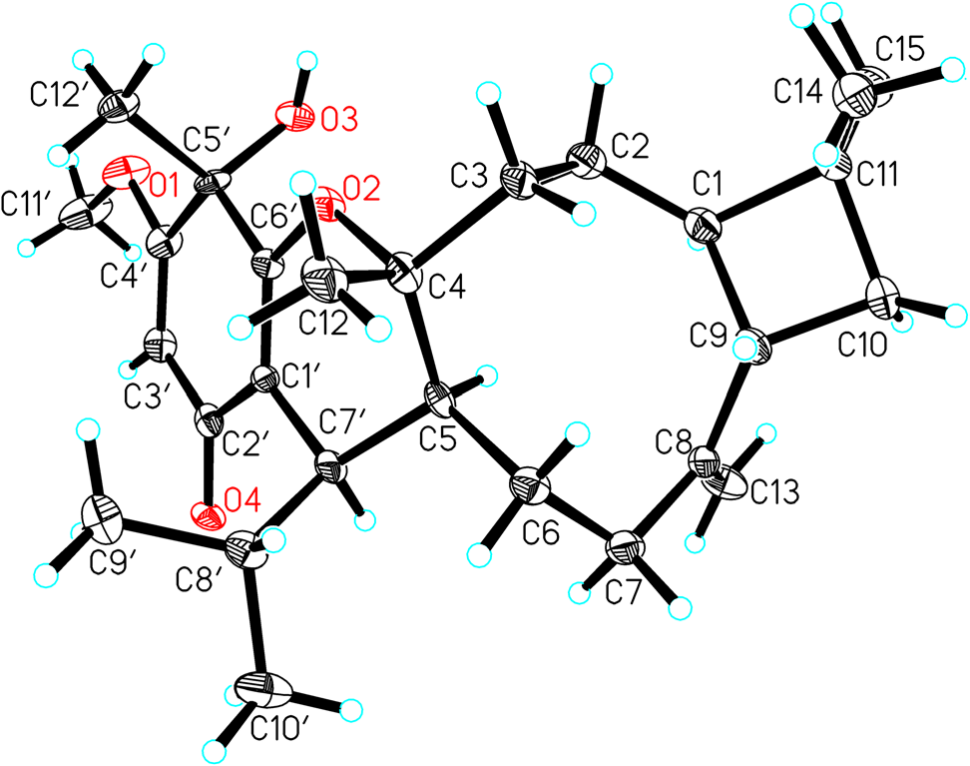
X-ray ORTEP drawing of **4**

In the NOESY spectrum, the correlations between H-5 and H-1/H-7′, between H-1 and Me-15, between H-9 and Me-14, as well as between Me-12 and H-8′/Me-12′ suggested that the relative configurations of C-1, C-4, C-5, C-9, and C-7′ were identical to those of myrtucommulone K (Fig. 3). Additionally, the structure and absolute configuration of **4** was unambiguously determined by X-ray crystallographic analysis using Cu K*α* radiation with the Flack parameter [0.08 (13)] (Fig. 5). Hence, the absolute configuration of **4** was defined as 1*R*, 4*R*, 5*S*, 9*S*, 5′*S* and 7′*R*.

### Bioactivity Evaluation

The antibacterial activities of compounds **1**–**4** against Gram-positive strains *Staphylococcus aureus* ATCC 43300 (MRSA), *S. aureus* ATCC 700699 (VISA), *S. aureus* ATCC 25923 and *Enterococcus faecalis* ATCC 29212 and Gram-negative strains *Pseudomonas aeruginosa* ATCC 27853, *Escherichia coli* ATCC 25922 and *Klebsiella pneumoniae* ATCC 700603 were measured by broth microdilution method. As a result, compound **4** exhibited moderate antibacterial activity against all Gram-positive strains with MIC value of 32 μg/mL (Table 2).

**Table 2:** Antibacterial activities of compounds **1**–**4** (MIC, μg/mL)

Microorganism	**1**	**2**	**3**	**4**	Ciprofloxacin^a^	Vancomycin^a^
*S. aureus* ATCC 43300 (MRSA)	> 128	> 128	> 128	32	–^b^	1
*S. aureus* ATCC 700699 (VISA)	> 128	> 128	> 128	32	–	8
*S. aureus* ATCC 25923	> 128	> 128	> 128	32	–	1
*E. faecalis* ATCC 29212	> 128	> 128	> 128	32	–	2
*P. aeruginosa* ATCC 27853	> 128	> 128	> 128	> 512	0.25	–
*E. coli* ATCC 25922	> 128	> 128	> 128	> 128	< 0.0625	–
*K. pneumoniae* ATCC 700603	> 128	> 128	> 128	> 128	0.5	–

^a^As positive controls

^b^NA

## Experimental

### General Methods

Melting points were obtained on a Buchi melting point B-545 apparatus (Buchi Instrument, Switzerland) and are uncorrected. Optical rotations were measured on a JASCO P-2000 digital polarimeter (Jasco Co., Ltd., Tokyo, Japan) at room temperature. IR spectra were determined on a JASCO FT/IR-4600 plus Fourier transform infrared spectrometer (Jasco Co., Ltd., Tokyo, Japan) using KBr pellets. UV spectra were recorded on a JASCO V-550 UV/Vis spectrophotometer (Jasco Co., Ltd., Tokyo, Japan). CD spectra were obtained on a ChirascanqCD (Applied Photophysics Ltd., Surrey, UK). HRESIMS spectra were acquired on an Agilent 6210 LC/MSD TOF mass spectrometer (Agilent Technologies, CA, USA). NMR spectra were measured on Bruker AV-500 or AV-400 spectrometers (Bruker, Switzerland) with TMS as internal standard, and chemical shifts were denoted in *δ* values (ppm). X-ray crystallographic analyses were carried out on an Agilent Gemini S Ultra CCD diffractometer with Cu K*α* radiation (*λ* = 1.54178 Å). Silica gel (200–300 mesh; Qingdao Marine Chemical, Inc., Qingdao, People’s Republic of China), Sephadex LH-20 (Pharmacia Biotech AB, Uppsala, Sweden), and reversed-phase C_18_ silica gel (YMC, Kyoto, Japan) were used for column chromatography (CC). Preparative HPLC was carried out on an Agilent 1260 Chromatograph equipped with a G1311C pump and a G1315D photodiode array detector (Agilent Technologies, CA, USA) with a semi-preparative C_18_ reversed-phase column (Cosmosil, 10 mm × 250 mm, 5 μm). All solvents used in CC and HPLC were of analytical grade (Shanghai Chemical Plant, Shanghai, People’s Republic of China) and chromatographic grade (Fisher Scientific, New Jersey, USA), respectively.

### Plant Material

The leaves of *M. cauliflora* were collected from Nanning city, Guangxi Province of People’s Republic of China, in July of 2018. A voucher specimen (No. 2018070607) identified by Professor Guang-Xiong Zhou (Jinan University) was deposited in the Institute of Traditional Chinese Medicine and Natural Products, Jinan University, Guangzhou, People’s Republic of China.

### Extraction and isolation

The air-dried leaves of *M. cauliflora* (15 kg) were powdered and extracted with 95% EtOH (v/v, 50 L) at room temperature. The extract (2.2 kg) was suspended in H_2_O and extracted with petroleum ether (PE, b.p. 60–90 °C). The PE extract (673.2 g) was subjected to a silica gel column chromatography using cyclohexane–EtOAc (100:0 → 0:100, v/v) as eluent to afford 10 fractions (Frs. A–J). Fr. G (48.3 g) was further separated by silica gel column using a gradient cyclohexane–EtOAc (100:0 → 0:100, v/v) to give 8 subfractions (Frs. G1–G8). Subfraction G5 (10.7 g) was chromatographed on Sephadex LH-20 (CH_2_Cl_2_/MeOH, 1:1, v/v) to obtain three subfractions (Frs. G5A–G5C). Subfraction G5B (7.3 g) was subjected to ODS column using MeOH/H_2_O (50:50 → 100:0, v/v) and further purified by semi-preparative reversed-phase HPLC (MeOH/H_2_O, 70:30, v/v, 3 mL/min) to afford **1** (12.5 mg, *t*_R_ 41.8 min), **3** (11.3 mg, *t*_R_ 33.7 min) and **4** (15.7 mg, *t*_R_ 49.3 min). Subfraction G6 (5.3 g) was separated on Sephadex LH-20 (CH_2_Cl_2_/MeOH, 1:1, v/v) to obtain **2** (7.3 mg).

***Compound 1*** yellow gum (CH_3_OH); [*α*]_D_^25^ =  + 119 (*c* = 0.50, MeOH); UV (MeOH) *λ*_max_ (log *ε*) 206 (3.73) nm; IR (KBr) *ν*_max_ 3475, 2977, 2954, 2876, 1611, 1584, 1488, 1445, 1415, 1385, 1307, 1236, 1199, 1132, 1084, 1035, 1014, 982, 904, 831 cm^−1^; ^1^H NMR (CDCl_3_, 500 MHz) and ^13^C NMR (CDCl_3_, 125 MHz), see Table 1; HRESIMS *m*/*z* 345.2423 [M+H]^+^ (calcd for C_22_H_33_O_3_: 345.2424); ECD (MeCN, Δ*ε*) 211 (+ 6.2), 238 (− 0.5), 277 (+ 0.7) nm.

***Compound 2*** yellow oil (CH_3_OH); [*α*]_D_^25^ =  + 59 (*c* = 0.50, MeOH); UV (MeOH) *λ*_max_ (log *ε*) 202 (3.85), 245 (3.92), 296 (3.50) nm; IR (KBr) *ν*_max_ 3357, 2957, 2933, 2871, 1656, 1605, 1462, 1385, 1353, 1236, 1137, 1092, 998, 983, 893, 843 cm^−1^; ^1^H NMR (CDCl_3_, 400 MHz) and ^13^C NMR (CDCl_3_, 100 MHz), see Table 1; HRESIMS *m*/*z* 361.2386 [M+H]^+^ (calcd for C_22_H_33_O_4_: 361.2373); ECD (MeCN, Δ*ε*) 203 (− 19.8), 244 (+ 11.7), 296 (+ 8.1), 337 (− 1.8) nm.

***Compound 3*** yellow oil (CH_3_OH); [*α*]_D_^25^ =  − 63 (*c* = 0.50, MeOH); UV (MeOH) *λ*_max_ (log *ε*) 202 (3.71), 245 (3.77), 297 (3.32) nm; IR (KBr) *ν*_max_ 3359, 2958, 2924, 2871, 1656, 1603, 1463, 1388, 1372, 1259, 1232, 1136, 1089, 989, 899, 842 cm^−1^; ^1^H NMR (CDCl_3_, 400 MHz) and ^13^C NMR (CDCl_3_, 100 MHz), see Table 1; HRESIMS *m*/*z* 361.2391 [M+H]^+^ (calcd for C_22_H_33_O_4_: 361.2373); ECD (MeCN, Δ*ε*) 203 (+ 18.0), 245 (− 20.5), 293 (− 9.6), 333 (+ 2.5) nm.

***Compound 4*** colorless blocks (CH_3_OH); m.p. 162–163 ℃; [*α*]_D_^25^ =  − 42 (*c* = 0.50, MeOH); UV (MeOH) *λ*_max_ (log *ε*) 205 (3.86), 247 (3.87), 310 (3.44) nm; IR (KBr) *ν*_max_ 3336, 2957, 2870, 1663, 1615, 1462, 1386, 1364, 1284, 1259, 1233, 1176, 1140, 999, 941, 886, 844 cm^−1^; ^1^H NMR (CDCl_3_, 500 MHz) and ^13^C NMR (CDCl_3_, 125 MHz), see Table 1; HRESIMS *m*/*z* 429.2996 [M + H]^+^ (calcd for C_27_H_41_O_4_: 429.2999).

### X-Ray Analysis

*Crystal data for 4* C_27_H_40_O_4_, monoclinic, space group *P*2_1_, *a* = 10.2148 (3) Å, *b* = 24.2552 (4) Å, *c* = 20.4139 (5) Å, *α* = 90°, *β* = 97.254 (2)°, *γ* = 90°, *V* = 5017.3 (2) Å^3^, *T* = 293 (2) K, *Z* = 2, *D*_calcd_ = 1.135 g cm^−3^, *F*(000) = 1872, 20761 reflections measured (2.18° ≤ *θ* ≤ 73.86°), 13408 unique (*R*_int_ = 0.0389, *R*_sigma_ = 0.0574) which were used in all calculations. The final *R*_1_ was 0.0916 [*I* > 2*σ*(*I*)] and w*R*_2_ was 0.2884 (all data). CCDC-1998470 contains the supplementary crystallographic data for this paper. These data can be obtained free of charge from The Cambridge Crystallographic Data Centre via http://www.ccdc.cam.ac.uk/data_request/cif.

### Antibacterial Activity Assay

*Staphylococcus aureus* ATCC 43300 (methicillin-resistant *S. aureus*, MRSA), *S. aureus* ATCC 700699 (vancomycin-intermediate *S. aureus*, VISA), *S. aureus* ATCC 25923, *E. faecalis* ATCC 29212, *P. aeruginosa* ATCC 27853, *E. coli* ATCC 25922 and *K. pneumoniae* ATCC 700603 were standard isolates from ATCC (Manassas, VA, USA). The MIC values were measured using a previously reported method [7]. Ciprofloxacin and vancomycin were used as positive controls.

## Electronic supplementary material

(PDF 2585 kb)
