# Supplementary material for: Four New Phloroglucinol-Terpene Adducts from the Leaves of Myrciaria cauliflora
Source: Nat Prod Bioprospect. 2020 Dec 6;11(1):111–8. doi: 10.1007/s13659-020-00288-4 (PMC7933295; doi:10.1007/s13659-020-00288-4)
Supplement: Supplementary file 1 — (PDF 2585 kb) [file 13659_2020_288_MOESM1_ESM.pdf]

Supplementary Material

Four new phloroglucinol-terpene adducts from the leaves of *Myrciaria cauliflora*

Ming Chen [a,b,c,§], Jia-Qing Cao [b,c,§], Wen-Jing Wang [b,c], Ni-Ping Li [b,c], Yan Wu [b,c], Lei Wang [b,c,*], Wen-Cai Ye [a,b,c,*]

[a] *Department of Natural Medicinal Chemistry, China Pharmaceutical University, Nanjing 210009, People's Republic of China*

[b] *Institute of Traditional Chinese Medicine & Natural Products, College of Pharmacy, Jinan University, Guangzhou 510632, People's Republic of China*

[c] *Guangdong Province Key Laboratory of Pharmacodynamic Constituents of TCM and New Drugs Research, Jinan University, Guangzhou 510632, People's Republic of China*

[§]Ming Chen and Jia-Qing Cao contributed equally to this work.

[*] Corresponding authors. Tel./fax: + 86 20 85221559.
E-mail addresses: chywc@aliyun.com (W.-C. Ye), cpuwanglei@126.com (L. Wang)

List of Supplementary Material

Contents: **page**

1. Single crystal X-ray data of **4**	3-4
2. Calculation details for **1**–**3**	5-24
Figure S4. UV spectrum of **1** in CH_3OH	25
Figure S5. IR (KBr disc) spectrum of **1**	25
Figure S6. HRESIMS spectrum of **1** in CH_3OH	26
Figure S7-13. 1D and 2D NMR spectra of **1** in $CDCl_3$	26-29
Figure S14. UV spectrum of **2** in CH_3OH	30
Figure S15. IR (KBr disc) spectrum of **2**	30
Figure S16. HRESIMS spectrum of **2** in CH_3OH	31
Figure S17-23. 1D and 2D NMR spectra of **2** in $CDCl_3$	31-34
Figure S24. UV spectrum of **3** in CH_3OH	35
Figure S25. IR (KBr disc) spectrum of **3**	35
Figure S26. HRESIMS spectrum of **3** in CH_3OH	36
Figure S27-33. 1D and 2D NMR spectra of **3** in $CDCl_3$	36-39
Figure S34. UV spectrum of **4** in CH_3OH	40
Figure S35. IR (KBr disc) spectrum of **4**	40
Figure S36. HRESIMS spectrum of **4** in CH_3OH	41
Figure S37-43. 1D and 2D NMR spectra of **4** in $CDCl_3$	41-44

1. Single crystal X-ray data of 4

X-ray Crystallographic Analysis. The single crystal of **4** was obtained from MeOH. X-ray diffraction data was collected on an Agilent Gemini S Ultra CCD diffractometer using Cu K*α* radiation (λ = 1.54178 Å). The structure was solved by direct methods and refined by full-matrix least squares on F_2 using the SHELXL-97 program. The crystallographic data have been deposited at the Cambridge Crystallographic Data Centre (CCDC 1998470).

***Table S1.* Crystal data and structure refinement for 4**

Empirical formula	$C_{27}H_{40}O_4$
Formula weight	428.59
Temperature	293 (2) K
Wavelength	1.54178 Å
Crystal system, Space group	Monoclinic, $P2_1$
Unit cell dimensions	a = 10.2148(3) Å, alpha = 90°
	b = 24.2552(4) Å, beta = 97.254(2)°
	c = 20.4139(5) Å, gamma = 90°
Volume	5017.3(2) $Å^3$
Z, Calculated density	2, 1.135 Mg/m^3
Absorption coefficient	0.585 mm^{-1}
F(000)	1872.0
Crystal size	0.13 × 0.12 × 0.11mm^3
Theta range for data collection	2.182 to 73.857°
Limiting indices	-12<=h<=10, -22<=k<=29, -25<=l<=24
Reflections collected / unique	20761/13408 [R(int) = 0.0389]
Max. and min. transmission	1.000 and 0.973
Data/restraints/parameters	13408/1/1149
Goodness-of-fit on F^2	1.091

Final R indexes [I>=2sigma(I)]	R_1 = 0.0916, wR_2 = 0.2833
R indexes (all data)	R_1 = 0.0983, wR_2 = 0.2884
Absolute structure parameter	0.08(13)
Largest diff. peak and hole	0.721 and -0.414 e. $Å^{-3}$

2. Chemical calculation details for 1-3

2.1 Chemical calculation details for 1

The conformational analysis of compound **1** was performed in the SYBYL 8.1 program by using MMFF94s molecular force field, which afforded 8 conformers of **1**, with an energy cutoff of 10 kcal mol^{-1} to the global minima. All the obtained conformers were further optimized using DFT at the CAM-B3LYP/6-31+G(d) level in acetonitrile by using Gaussian09 software,[1] and 2 conformers of **1** were selected. All of the optimized stable conformers were used for TDDFT computation of the excited stats at the same levels, with the consideration of the first 50 excitations. The overall ECD curves of **1** were weighted by Boltzmann distribution of each conformer (with a half-bandwidth of 0.25 eV), with a UV correction of 11 nm. The calculated ECD spectra of **1** were subsequently compared with the experimental one. The ECD spectra were produced by SpecDis 1.6 software.[2]

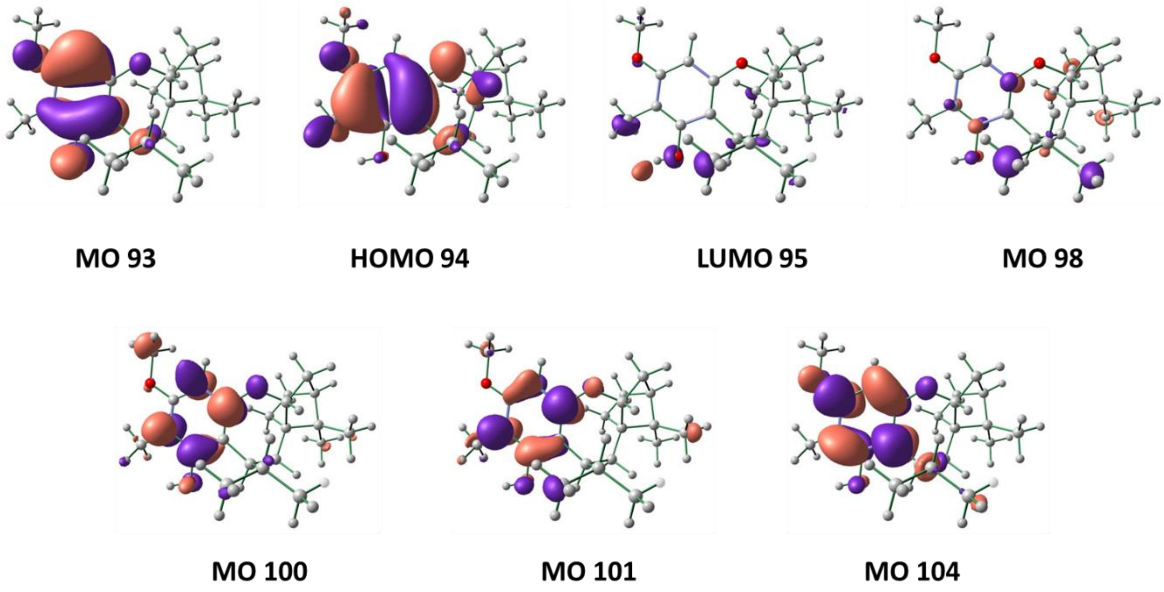

Figure S1. Key molecular orbitals involved in important transitions regarding the ECD spectra of predominant conformer of **1**.

Table S2. Key transitions and their related rotatory and oscillator strengths of predominant conformer of **1**.

**HOMO is 94**					
**No.**	**Energy (cm-**	**Wavelength**	**Osc.**	**R(length)**	**Major contribs**

	[1])	(nm)	**Strength**		
1	41310.39008	242.0698517	0.0075	-2.2449	H-1->L+6 (11%), H-1->L+9 (15%), HOMO->L+5 (11%), HOMO->L+9 (15%), HOMO->L+10 (11%)
2	43932.51664	227.6218338	0.007	-3.2239	HOMO->LUMO (67%)
3	45455.30192	219.9963388	0.0751	12.8955	HOMO->L+5 (16%), HOMO->L+6 (19%), HOMO->L+9 (21%)
4	46081.19248	217.008273	0.0112	2.2407	H-1->LUMO (66%), H-1->L+1 (13%)
5	48210.51088	207.4236472	0.0045	8.2375	HOMO->L+1 (28%), HOMO->L+2 (40%)
6	49300.98	202.8357246	0.3738	-24.4527	H-1->L+3 (13%), H-1->L+5 (16%), H-1->L+6 (12%), HOMO->L+3 (10%), HOMO->L+9 (18%)
7	50011.55936	199.9537732	0.086	-81.1697	H-1->L+2 (47%)
8	50549.53488	197.8257569	0.1413	-34.6704	HOMO->L+3 (38%)
9	50812.47344	196.8020709	0.6847	114.289	H-1->L+9 (34%)
10	50982.6576	196.1451299	0.0997	-6.0181	HOMO->LUMO (10%), HOMO->L+1 (19%), HOMO->L+5 (13%)
11	52664.3352	189.8818235	0.0004	1.5149	HOMO->L+7 (39%)
12	53145.85152	188.1614409	0.0048	-6.8704	H-1->L+8 (10%), HOMO->L+4 (10%), HOMO->L+6 (14%)
13	53662.04992	186.3514349	0.0788	6.8832	H-1->L+3 (22%), H-1->L+8 (23%)
14	53860.46368	185.6649445	0.0247	12.3855	H-1->L+3 (10%), H-1->L+7 (10%), H-1->L+10 (12%), HOMO->L+6 (11%)

15	54149.21216	184.6748937	0.0056	-1.9584	HOMO->L+4 (13%), HOMO->L+5 (20%)
16	54624.276	183.068788	0.0022	-3.1022	H-1->L+1 (13%), H-1->L+7 (19%)
17	55370.344	180.6020927	0.0265	-2.4673	HOMO->L+4 (28%), HOMO->L+8 (14%)
18	55953.48688	178.7198718	0.0086	-5.0785	HOMO->L+3 (16%), HOMO->L+7 (13%), HOMO->L+10 (24%)
19	56814.89296	176.0101882	0.0013	2.9724	H-1->L+4 (29%)
20	57110.90048	175.097922	0.0038	6.0361	H-1->L+5 (19%), H-1->L+6 (13%), H-1->L+11 (14%)
21	57314.96016	174.4745172	0.0008	0.5595	HOMO->L+8 (20%), HOMO->L+11 (29%)
22	58041.67072	172.2900095	0.0008	2.62	H-1->L+7 (12%), H-1->L+10 (17%), HOMO->L+13 (13%)
23	58245.7304	171.686404	0.0049	2.096	HOMO->L+12 (15%), HOMO->L+13 (27%)
24	58693.3712	170.3769914	0.0038	0.6528	HOMO->L+12 (32%), HOMO->L+13 (20%)
25	58810.3224	170.0381768	0.0006	-1.1289	H-1->L+3 (21%), H-1->L+4 (14%), H-1->L+14 (11%), HOMO->L+12 (12%)
26	59622.52832	167.7218374	0.0118	13.2166	HOMO->L+14 (31%), HOMO->L+15 (19%)
27	60103.23808	166.3803868	0.0008	0.8283	HOMO->L+16 (29%), HOMO->L+17 (11%)
28	60283.90752	165.8817487	0.0024	-2.8605	H-1->L+8 (10%), H-1->L+11 (11%), H-1-

					>L+13 (15%)
29	60635.56768	164.9197061	0.0037	-3.6992	H-1->L+11 (16%)
30	60700.09248	164.7443948	0.0021	-2.4891	HOMO->L+14 (20%), HOMO->L+15 (24%)
31	60935.608	164.1076594	0.0032	3.4049	HOMO->L+15 (11%)
32	61582.46912	162.3838755	0.0079	2.1599	H-1->L+12 (33%), H-1->L+13 (24%)
33	61998.65408	161.293824	0.0196	-3.4123	H-1->L+13 (12%), HOMO->L+17 (20%)
34	62246.268	160.6522017	0.0852	-15.9268	H-2->LUMO (14%), H-2->L+9 (18%)
35	62390.64224	160.2804466	0.0156	8.8333	H-1->L+16 (14%)
36	62643.90208	159.6324569	0.0412	3.5142	H-2->LUMO (12%), H-2->L+9 (12%)
37	62698.74816	159.4928175	0.0142	5.1448	H-3->LUMO (23%), H-3->L+1 (12%), HOMO->L+20 (18%)
38	62961.68672	158.8267488	0.005	3.2459	HOMO->L+19 (12%), HOMO->L+20 (14%)
39	63074.60512	158.5424115	0.0047	-1.0031	HOMO->L+18 (32%)
40	63177.8448	158.2833354	0.009	-4.1062	H-1->L+14 (24%), H-1->L+15 (28%)
41	63416.58656	157.6874528	0.0115	-8.0608	H-1->L+10 (12%), H-1->L+14 (21%), H-1->L+15 (11%), H-1->L+21 (12%)
42	63816.64032	156.6989417	0	0.5604	H-2->L+1 (10%), HOMO->L+21 (13%)
43	64177.17264	155.8186437	0.002	-1.1172	HOMO->L+21 (17%), HOMO->L+23 (16%)

44	64410.26848	155.254748	0.0066	-12.9386	
45	64500.6032	155.0373098	0.0163	-12.2424	
46	64572.38704	154.8649579	0.0067	-10.4619	H-2->L+1 (14%), HOMO->L+20 (11%)
47	64776.44672	154.3771001	0.0023	-2.6853	H-1->L+17 (19%)
48	65162.78896	153.4618171	0.034	12.1512	H-3->L+1 (10%), H-3->L+2 (30%), H-2->L+2 (13%)
49	65299.0976	153.1414731	0.0009	0.5767	H-1->L+20 (23%)
50	65520.9016	152.6230524	0.0003	0.6816	HOMO->L+24 (20%)

Table S3. Cartesian coordinates of predominant conformer of **1**.

**Standard orientation:**					
**Center Number**	**Atomic Number**	**Atomic Type**	**Coordinates(Angstroms)**		
			**X**	**Y**	**Z**
1	6	0	-3.22518	-0.94759	-0.13474
2	6	0	-3.32337	0.185434	0.684457
3	6	0	-2.18572	0.980647	0.802245
4	6	0	-0.96036	0.723981	0.15837
5	6	0	-0.91419	-0.43741	-0.61766
6	6	0	-2.03582	-1.26144	-0.77166
7	6	0	0.178978	1.722329	0.287784
8	6	0	1.433185	1.096446	0.943714
9	6	0	1.884885	-0.27651	0.433792
10	6	0	1.52937	-0.55012	-1.04008
11	8	0	0.169473	-0.91208	-1.30433
12	6	0	1.380268	-1.49299	1.258426
13	6	0	2.334554	-2.65111	0.861811

14	6	0	3.271091	-1.98283	-0.1592
15	6	0	2.444258	-1.7357	-1.43496
16	6	0	0.376879	2.515588	-1.04031
17	6	0	-0.87811	3.341394	-1.34635
18	6	0	1.595139	3.441858	-1.00407
19	6	0	3.433609	-0.5497	0.412088
20	6	0	4.08934	-0.49127	1.797391
21	8	0	-2.23463	2.109353	1.581131
22	6	0	-4.59975	0.536658	1.403154
23	8	0	-4.35375	-1.70697	-0.24492
24	6	0	4.255998	0.377716	-0.49436
25	6	0	-4.31549	-2.85893	-1.06237
26	1	0	-1.92274	-2.14227	-1.38897
27	1	0	-0.15619	2.478467	1.002333
28	1	0	1.222983	1.010151	2.01624
29	1	0	2.258679	1.8113	0.866237
30	1	0	1.753619	0.324446	-1.65258
31	1	0	1.441462	-1.26492	2.32663
32	1	0	0.336028	-1.72937	1.053734
33	1	0	1.80046	-3.49795	0.419835
34	1	0	2.885788	-3.03698	1.72479
35	1	0	4.20633	-2.52503	-0.33275
36	1	0	3.071866	-1.48454	-2.2948
37	1	0	1.839372	-2.6025	-1.71409
38	1	0	0.504517	1.814494	-1.87278
39	1	0	-1.7717	2.714541	-1.41145
40	1	0	-1.0516	4.090534	-0.56457
41	1	0	-0.76781	3.868727	-2.30031

42	1	0	1.613303	4.080389	-1.89411
43	1	0	2.542103	2.895767	-0.97706
44	1	0	1.561443	4.100614	-0.12675
45	1	0	5.136622	-0.80828	1.729213
46	1	0	4.084998	0.535514	2.180822
47	1	0	3.604097	-1.12156	2.54388
48	1	0	-3.11567	2.200572	1.968285
49	1	0	-4.99904	1.511217	1.088843
50	1	0	-5.36978	-0.20551	1.192379
51	1	0	-4.46757	0.561692	2.493844
52	1	0	3.88978	0.44704	-1.52017
53	1	0	5.290779	0.019289	-0.5433
54	1	0	4.28922	1.393967	-0.08677
55	1	0	-5.31424	-3.2939	-1.01263
56	1	0	-4.08051	-2.60153	-2.10173
57	1	0	-3.58276	-3.58562	-0.69217

2.2 Chemical calculation details for 2

The conformational analysis of compound **2** was performed in the SYBYL 8.1 program by using MMFF94s molecular force field, which afforded 10 conformers of **2**, with an energy cutoff of 10 kcal mol^{-1} to the global minima. All the obtained conformers were further optimized using DFT at the CAM-B3LYP/6-31+G(d) level in acetonitrile by using Gaussian09 software,[1] and 3 conformers of **2** were selected. All of the optimized stable conformers were used for TDDFT computation of the excited stats at the same levels, with the consideration of the first 50 excitations. The overall ECD curves of **2** were weighted by Boltzmann distribution of each conformer (with a half-bandwidth of 0.4 eV), with a UV correction of 15 nm. The calculated ECD spectra of **2** were subsequently compared with the experimental one. The ECD spectra were produced by SpecDis 1.6 software.[2]

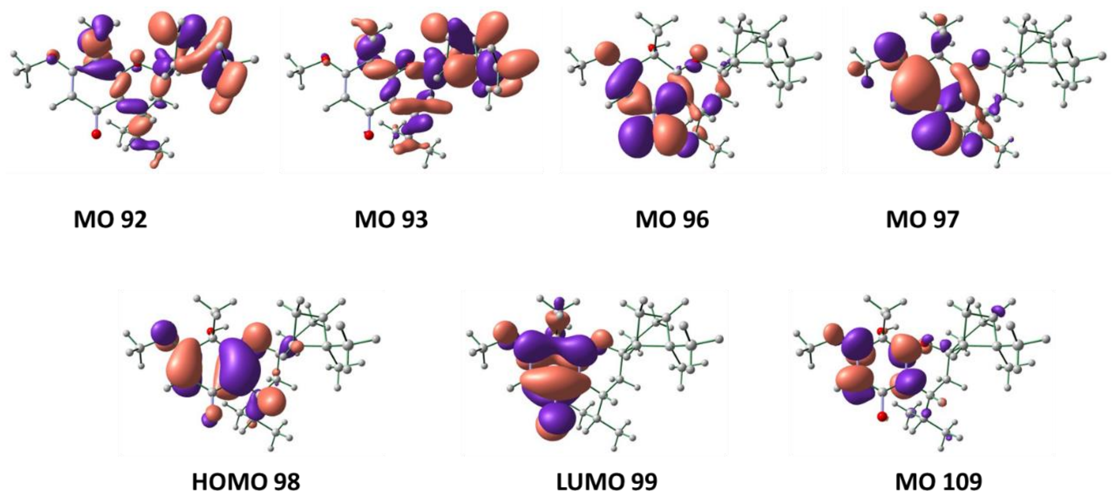

Figure S2. Key molecular orbitals involved in important transitions regarding the ECD spectra of predominant conformer of **2**.

Table S4. Key transitions and their related rotatory and oscillator strengths of predominant conformer of **2**.

**HOMO is 98**					
**No.**	**Energy (cm^{-1})**	**Wavelength (nm)**	**Osc. Strength**	**R(length)**	**Major contribs**
1	33779.54	296.0372	0.0017	-9.6112	H-2->LUMO (66%), H-1->LUMO (23%)
2	35576.56	281.084	0.1373	37.1377	HOMO->LUMO (96%)
3	44003.49	227.2547	0.3489	23.9506	H-2->LUMO (23%), H-1->LUMO (69%)
4	49361.47	202.5872	0.0142	-11.8207	H-6->LUMO (41%), H-5->LUMO (34%)
5	51247.21	195.1326	0.032	-13.8028	HOMO->L+1 (63%)
6	53546.71	186.7528	0.1181	-9.2432	H-3->LUMO (11%), HOMO->L+8 (13%), HOMO->L+10 (24%)
7	54380.69	183.8888	0.0677	42.2367	H-7->LUMO (17%), H-3->LUMO (32%)
8	54635.57	183.031	0.0235	-22.9834	H-1->L+1 (15%), HOMO->L+4 (28%)
9	54878.34	182.2212	0.0077	-16.7186	H-1->L+1 (11%), HOMO->L+2 (14%),

					HOMO->L+4 (23%)
10	55488.91	180.2162	0.0872	-81.5991	H-10->LUMO (15%), H-9->LUMO (15%), H-4->LUMO (12%)
11	55601.83	179.8502	0.0322	-33.8553	H-2->L+1 (22%), H-2->L+3 (18%)
12	56390.64	177.3344	0.0239	18.7052	HOMO->L+2 (12%), HOMO->L+3 (16%), HOMO->L+4 (16%)
13	56969.75	175.5317	0.0434	-7.9901	H-5->LUMO (31%), H-4->LUMO (13%), H-3->LUMO (18%)
14	57254.47	174.6589	0.0111	9.6852	H-2->L+10 (12%), H-1->L+6 (10%), H-1->L+8 (10%), H-1->L+10 (19%)
15	57961.01	172.5298	0.0127	44.8524	H-2->L+8 (12%), H-2->L+10 (16%), HOMO->L+2 (13%)
16	58094.9	172.1321	0.0075	-10.6028	HOMO->L+2 (13%)
17	58545.77	170.8065	0.0111	-10.6672	HOMO->L+1 (11%), HOMO->L+2 (13%), HOMO->L+3 (28%)
18	59060.36	169.3183	0.0102	-2.3069	H-10->LUMO (21%), H-8->LUMO (13%), H-7->LUMO (12%), H-4->LUMO (17%)
19	59915.31	166.9023	0.0067	-9.6506	HOMO->L+5 (12%), HOMO->L+7 (12%)
20	60480.71	165.342	0.0031	-0.3387	H-12->LUMO (12%), H-8->LUMO (43%)
21	60579.11	165.0734	0.0041	0.7255	HOMO->L+7 (22%), HOMO->L+9 (26%)
22	60914.64	164.1642	0.0145	6.6759	H-6->LUMO (37%), H-5->LUMO (11%), H-4->LUMO (19%)
23	61045.3	163.8128	0.0604	-51.3497	H-3->L+1 (11%)
24	61196.93	163.4069	0.0052	-2.5794	H-3->L+1 (14%)

25	61299.37	163.1338	0.0129	14.7335	H-3->L+1 (25%)
26	61742.17	161.9639	0.0119	-3.4844	H-2->L+2 (11%), H-1->L+2 (13%)
27	61793.79	161.8286	0.0347	-58.7868	
28	62037.37	161.1932	0.0068	11.6611	H-2->L+4 (11%)
29	62224.49	160.7084	0.026	20.4915	HOMO->L+5 (15%), HOMO->L+8 (22%)
30	62431.78	160.1748	0.0198	-0.1	
31	62526.95	159.931	0.0167	-1.4902	
32	62836.67	159.1427	0.0082	6.3539	
33	63247.21	158.1097	0.0068	0.946	H-9->LUMO (16%), H-7->LUMO (13%)
34	63298.02	157.9828	0.0045	4.8791	H-9->LUMO (11%), H-7->LUMO (10%), HOMO->L+9 (10%)
35	63862.61	156.5861	0.0305	39.2158	HOMO->L+6 (14%), HOMO->L+10 (11%)
36	64007.8	156.231	0.004	8.5907	H-4->L+1 (12%), HOMO->L+11 (14%)
37	64344.13	155.4143	0.0349	57.2456	
38	64445.76	155.1693	0.0113	18.3545	H-11->LUMO (13%), H-4->L+1 (11%)
39	64527.22	154.9734	0.0175	-28.6634	H-3->L+2 (36%)
40	64820.81	154.2715	0.0056	-12.1909	HOMO->L+12 (16%)
41	64963.57	153.9324	0.0105	-4.7675	H-11->LUMO (12%), H-5->L+1 (12%)
42	65077.29	153.6634	0.0115	7.0832	H-11->LUMO (20%)
43	65363.62	152.9903	0.0099	-22.4962	
44	65491.87	152.6907	0.0163	21.8983	H-3->L+4 (15%), HOMO->L+15 (12%)
45	65561.23	152.5292	0.0218	-13.2939	H-5->L+1 (11%)
46	65708.83	152.1865	0.0075	-19.3593	HOMO->L+13 (11%)
47	65786.26	152.0074	0.0046	2.4489	HOMO->L+11 (12%)

48	65958.86	151.6096	0.0014	10.6883	H-3->L+5 (11%)
49	66285.52	150.8625	0.0149	-13.9892	
50	66385.53	150.6352	0.0159	-40.1543	

Table S5. Cartesian coordinates of predominant conformer of **2**.

**Standard orientation:**					
**Center Number**	**Atomic Number**	**Atomic Type**	**Coordinates (Angstroms)**		
			**X**	**Y**	**Z**
1	6	0	3.327208	-0.80314	0.178436
2	6	0	3.370133	0.346007	0.87068
3	6	0	2.180164	1.187261	1.043805
4	6	0	0.96293	0.848594	0.27841
5	6	0	0.907438	-0.36166	-0.30316
6	6	0	2.040698	-1.36897	-0.37347
7	6	0	-0.19621	1.819001	0.199682
8	6	0	-1.24696	1.278513	-0.77961
9	6	0	-1.47522	-0.22817	-0.6852
10	8	0	-0.17741	-0.86486	-0.93661
11	6	0	-2.40262	-0.74082	-1.81757
12	6	0	-3.55809	-1.69266	-1.38988
13	6	0	-3.61699	-1.91359	0.122312
14	6	0	-3.51112	-0.567	0.894711
15	6	0	-1.96108	-0.71678	0.684955
16	6	0	0.240423	3.264903	-0.17154
17	6	0	1.027366	3.35932	-1.48137
18	6	0	-0.95665	4.219764	-0.19032
19	8	0	4.357941	-1.63025	-0.04497
20	8	0	2.213708	2.152176	1.805254

21	6	0	5.641828	-1.23574	0.410432
22	8	0	2.27514	-1.69068	-1.74172
23	6	0	1.681321	-2.63901	0.412989
24	6	0	-2.19185	-2.24722	0.606781
25	6	0	-3.8602	-0.72318	2.379244
26	6	0	-4.2952	0.632678	0.366065
27	1	0	4.268388	0.712306	1.351649
28	1	0	-0.64565	1.895047	1.200073
29	1	0	-0.93098	1.485205	-1.8088
30	1	0	-2.19493	1.79622	-0.63198
31	1	0	-1.7621	-1.23558	-2.55213
32	1	0	-2.82164	0.128782	-2.33227
33	1	0	-4.51397	-1.29466	-1.74823
34	1	0	-3.43168	-2.66771	-1.87407
35	1	0	-4.43789	-2.58684	0.395892
36	1	0	-1.32132	-0.33737	1.486988
37	1	0	0.904773	3.586949	0.633126
38	1	0	1.389195	4.383182	-1.62579
39	1	0	1.899681	2.698289	-1.48279
40	1	0	0.415435	3.107636	-2.35541
41	1	0	-1.54994	4.138611	0.728369
42	1	0	-1.62271	4.033504	-1.04092
43	1	0	-0.61321	5.2568	-0.2699
44	1	0	6.326763	-2.01792	0.084913
45	1	0	5.931038	-0.27753	-0.034
46	1	0	5.659936	-1.1559	1.503024
47	1	0	1.410136	-1.87447	-2.14143
48	1	0	0.771378	-3.08152	0.002176

49	1	0	2.500616	-3.355	0.322963
50	1	0	1.513207	-2.41342	1.47085
51	1	0	-1.55682	-2.80582	-0.085
52	1	0	-2.15753	-2.73365	1.582343
53	1	0	-3.59018	0.183783	2.932692
54	1	0	-4.93846	-0.87831	2.502094
55	1	0	-3.34971	-1.56324	2.856184
56	1	0	-4.16908	0.811607	-0.70268
57	1	0	-5.36565	0.47075	0.541815
58	1	0	-4.01615	1.550578	0.896276

2.3 Chemical calculation details for 3

The conformational analysis of compound **3** was performed in the SYBYL 8.1 program by using MMFF94s molecular force field, which afforded 25 conformers of **3**, with an energy cutoff of 10 kcal mol^{-1} to the global minima. All the obtained conformers were further optimized using DFT at the B3LYP/6-31+G(d) level in acetonitrile by using Gaussian09 software,[1] and 6 conformers of **3** were selected. All of the optimized stable conformers were used for TDDFT computation of the excited stats at the same levels, with the consideration of the first 50 excitations. The overall ECD curves of **3** were weighted by Boltzmann distribution of each conformer (with a half-bandwidth of 0.3 eV), with a UV correction of 15 nm. The calculated ECD spectra of **3** were subsequently compared with the experimental one. The ECD spectra were produced by SpecDis 1.6 software.[2]

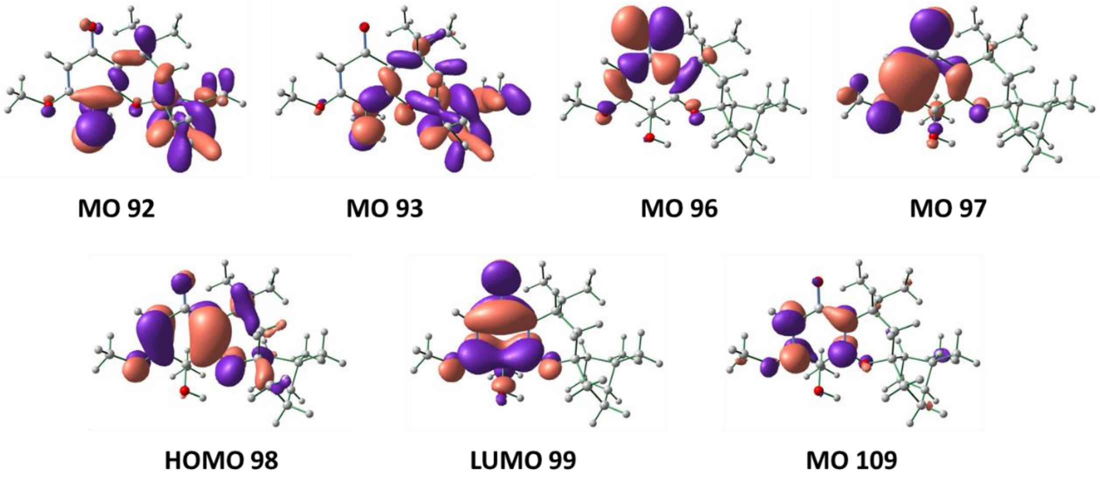

Figure S3. Key molecular orbitals involved in important transitions regarding the ECD spectra of predominant conformer of **3**.

Table S6. Key transitions and their related rotatory and oscillator strengths of predominant conformer of **3**.

**HOMO is 98**					
**No.**	**Energy (cm-1)**	**Wavelength (nm)**	**Osc. Strength**	**R(length)**	**Major contribs**
1	34255.41	291.9247	0.007	18.9975	H-2->LUMO (86%)
2	35492.67	281.7483	0.1406	-35.6312	HOMO->LUMO (88%)
3	44318.86	225.6376	0.3828	-35.2036	H-1->LUMO (91%)
4	49290.49	202.8789	0.0049	6.5796	H-6->LUMO (48%), H-5->LUMO (38%)
5	50776.98	196.9396	0.0086	-3.8934	HOMO->L+1 (63%), HOMO->L+3 (10%)
6	53477.35	186.9951	0.1543	35.6228	H-7->LUMO (10%), H-3->LUMO (19%), HOMO->L+7 (14%), HOMO->L+10 (16%), HOMO->L+11 (14%)
7	54105.66	184.8236	0.0937	-10.6286	H-3->LUMO (46%)
8	54581.53	183.2122	0.018	-14.1848	H-1->L+1 (22%), H-1->L+3 (19%), HOMO->L+2 (10%), HOMO->L+5 (13%)

9	54791.23	182.5109	0.0272	31.156	HOMO->L+2 (16%), HOMO->L+3 (37%), HOMO->L+4 (13%)
10	55467.94	180.2843	0.0171	33.8649	H-13->LUMO (10%), H-10->LUMO (19%), H-9->LUMO (35%)
11	55982.52	178.6272	0.0218	-7.3526	H-2->L+3 (10%), HOMO->L+4 (32%)
12	56322.89	177.5477	0.02	-4.7169	H-2->L+1 (17%), H-2->L+3 (31%), HOMO->L+4 (12%)
13	57299.64	174.5212	0.0238	-11.1251	H-8->LUMO (11%), H-4->LUMO (26%)
14	57429.49	174.1266	0.0275	-2.1701	H-1->L+7 (19%), H-1->L+10 (11%)
15	58010.21	172.3834	0.0265	15.3775	H-4->LUMO (11%), H-2->L+7 (16%), H-2->L+10 (12%), H-2->L+11 (11%)
16	58062.64	172.2278	0.0074	15.2292	H-1->L+3 (12%), HOMO->L+1 (14%), HOMO->L+2 (27%), HOMO->L+3 (13%)
17	58249.76	171.6745	0.0193	-23.5634	H-1->L+11 (18%)
18	59538.65	167.9581	0.0053	-11.4958	HOMO->L+5 (10%), HOMO->L+8 (16%), HOMO->L+9 (14%)
19	59659.63	167.6175	0.0153	-14.459	H-12->LUMO (10%), H-9->LUMO (11%), H-7->LUMO (23%), H-3->LUMO (19%)
20	60367.79	165.6513	0.0225	-6.3152	HOMO->L+6 (12%), HOMO->L+7 (15%), HOMO->L+8 (19%)
21	60687.99	164.7772	0.0019	2.1712	HOMO->L+5 (10%), HOMO->L+7 (15%), HOMO->L+9 (36%)
22	61070.3	163.7457	0.0238	-1.6438	H-3->L+1 (62%), H-3->L+3 (17%)

23	61316.3	163.0888	0.0193	19.1547	H-8->LUMO (12%), H-4->LUMO (15%), HOMO->L+8 (10%)
24	61380.83	162.9173	0.0528	20.5207	HOMO->L+8 (13%)
25	61604.25	162.3265	0.0127	-37.8792	HOMO->L+6 (15%)
26	61921.22	161.4955	0.0097	5.9981	H-6->L+1 (10%)
27	62222.88	160.7126	0.0012	4.7038	H-16->LUMO (16%), H-12->LUMO (11%)
28	62381.77	160.3032	0.0112	17.5397	H-1->L+2 (11%)
29	62433.39	160.1707	0.0058	-0.4608	H-8->LUMO (27%), H-6->LUMO (11%), H-5->LUMO (11%)
30	62702.78	159.4826	0.0024	5.3258	H-8->LUMO (12%), H-2->L+4 (18%), H-2->L+7 (10%)
31	63018.15	158.6845	0.0048	4.5521	
32	63130.26	158.4026	0.0184	25.2101	H-2->L+1 (23%), H-2->L+5 (11%)
33	63532.73	157.3992	0.0027	3.6323	
34	63831.96	156.6613	0.0112	-14.3815	H-10->LUMO (28%), H-9->LUMO (15%)
35	63969.89	156.3236	0.008	-4.2166	H-4->L+1 (11%)
36	64214.27	155.7286	0.0222	-8.3958	H-3->L+2 (41%)
37	64404.62	155.2684	0.0255	-0.0963	H-3->L+2 (10%)
38	64445.76	155.1693	0.0097	6.8927	H-2->L+5 (10%)
39	64787.74	154.3502	0.0263	-23.3445	H-3->L+4 (14%), HOMO->L+12 (15%)
40	64820	154.2734	0.0306	-27.82	H-4->L+1 (14%), H-3->L+4 (29%)
41	64978.09	153.898	0.0042	6.4575	H-11->LUMO (19%), H-7->LUMO (14%)
42	65163.6	153.4599	0.0195	-9.2916	HOMO->L+13 (17%), HOMO->L+15 (12%)
43	65328.94	153.0715	0.0202	17.3161	H-5->L+1 (11%)

44	65447.5	152.7942	0.0191	7.8392	H-1->L+1 (11%)
45	65609.62	152.4167	0.0046	-1.8797	H-13->LUMO (10%), H-11->LUMO (18%)
46	65695.12	152.2183	0.008	-4.5617	HOMO->L+10 (15%), HOMO->L+14 (18%)
47	65791.91	151.9944	0.0176	-24.3139	H-3->L+1 (14%), H-3->L+3 (21%), H-3->L+5 (20%)
48	66044.36	151.4134	0.0137	-5.1447	HOMO->L+13 (19%), HOMO->L+16 (21%), HOMO->L+17 (14%)
49	66281.49	150.8717	0.0061	4.8573	H-1->L+6 (10%), H-1->L+12 (20%), HOMO->L+12 (13%)
50	66291.97	150.8478	0.0565	-5.2741	H-16->LUMO (19%), H-10->LUMO (10%)

Table S7. Cartesian coordinates of predominant conformer of **3**.

**Standard orientation:**					
**Center Number**	**Atomic Number**	**Atomic Type**	**Coordinates (Angstroms)**		
			**X**	**Y**	**Z**
1	6	0	3.542852	-0.66165	0.036191
2	6	0	3.707363	0.598763	-0.39617
3	6	0	2.567751	1.495462	-0.62712
4	6	0	1.213768	0.985135	-0.35177
5	6	0	1.066096	-0.29859	0.009704
6	6	0	2.190305	-1.24923	0.370988
7	6	0	-0.00692	1.823961	-0.61934
8	6	0	-1.04493	0.885299	-1.26178
9	6	0	-1.34535	-0.42216	-0.5106
10	8	0	-0.11781	-0.93363	0.126266
11	6	0	-1.79974	-1.52333	-1.5117

12	6	0	-2.96456	-2.44112	-1.04877
13	6	0	-3.64885	-1.92987	0.221182
14	6	0	-3.86276	-0.3884	0.176592
15	6	0	-2.36039	-0.29768	0.634796
16	6	0	-0.47465	2.625213	0.624898
17	6	0	-1.76019	3.40884	0.345691
18	6	0	0.613194	3.585884	1.114273
19	8	0	4.521655	-1.54833	0.270636
20	8	0	2.74726	2.629708	-1.06459
21	6	0	5.856942	-1.16048	-0.00759
22	8	0	2.058283	-2.44163	-0.39649
23	6	0	2.127965	-1.57676	1.871718
24	6	0	-2.55162	-1.69492	1.277918
25	6	0	-4.78468	0.095121	1.303314
26	6	0	-4.3739	0.243949	-1.11813
27	1	0	4.679623	1.022681	-0.61338
28	1	0	0.267007	2.567475	-1.37765
29	1	0	-1.99153	1.397513	-1.44326
30	1	0	-0.64709	0.618488	-2.24784
31	1	0	-2.08894	-1.02822	-2.44152
32	1	0	-0.91737	-2.11707	-1.76715
33	1	0	-3.69299	-2.53457	-1.86232
34	1	0	-2.59394	-3.45366	-0.85101
35	1	0	-4.51259	-2.55045	0.487329
36	1	0	-2.10461	0.529997	1.298107
37	1	0	-0.66871	1.917421	1.440478
38	1	0	-1.62943	4.073662	-0.5174
39	1	0	-2.02215	4.034343	1.205822

40	1	0	-2.61747	2.760355	0.142374
41	1	0	0.254454	4.151041	1.981807
42	1	0	1.522805	3.058544	1.412079
43	1	0	0.893786	4.297701	0.331169
44	1	0	6.15356	-0.31049	0.616921
45	1	0	6.474024	-2.02697	0.227766
46	1	0	5.970603	-0.89837	-1.06482
47	1	0	1.128799	-2.71183	-0.32532
48	1	0	1.152579	-2.00703	2.114085
49	1	0	2.910366	-2.29967	2.110278
50	1	0	2.270856	-0.67599	2.476686
51	1	0	-2.92629	-1.64059	2.300565
52	1	0	-1.69288	-2.36959	1.258351
53	1	0	-4.75462	1.188425	1.378905
54	1	0	-5.82147	-0.19378	1.095175
55	1	0	-4.52066	-0.30938	2.283065
56	1	0	-5.41199	-0.06811	-1.28532
57	1	0	-4.37654	1.337882	-1.0475
58	1	0	-3.80841	-0.02937	-2.00886

Reference

[1] Gaussian 09, Revision A.02, M. J. Frisch, G. W. Trucks, H. B. Schlegel, G. E. Scuseria, M. A. Robb, J. R. Cheeseman, G. Scalmani, V. Barone, B. Mennucci, G. A. Petersson, H. Nakatsuji, M. Caricato, X. Li, H. P. Hratchian, A. F. Izmaylov, J. Bloino, G. Zheng, J. L. Sonnenberg, M. Hada, M. Ehara, K. Toyota, R. Fukuda, J. Hasegawa, M. Ishida, T. Nakajima, Y. Honda, O. Kitao, H. Nakai, T. Vreven, J. A. Montgomery, Jr., J. E. Peralta, F. Ogliaro, M. Bearpark, J. J. Heyd, E. Brothers, K. N. Kudin, V. N. Staroverov, R. Kobayashi, J. Normand, K. Raghavachari, A. Rendell, J. C. Burant, S. S. Iyengar, J. Tomasi, M. Cossi, N. Rega, J. M. Millam, M. Klene, J. E. Knox, J. B. Cross, V. Bakken, C. Adamo, J. Jaramillo, R. Gomperts, R. E. Stratmann,

O. Yazyev, A. J. Austin, R. Cammi, C. Pomelli, J. W. Ochterski, R. L. Martin, K. Morokuma, V. G. Zakrzewski, G. A. Voth, P. Salvador, J. J. Dannenberg, S. Dapprich, A. D. Daniels, O. Farkas, J. B. Foresman, J. V. Ortiz, J. Cioslowski, and D. J. Fox, Gaussian, Inc., Wallingford CT, 2009.

[2] T. Bruhn, A. Schaumlöffel, Y. Hemberger, G. Bringmann, SpecDis version 1.60, University of Wuerzburg, Germany, 2012.

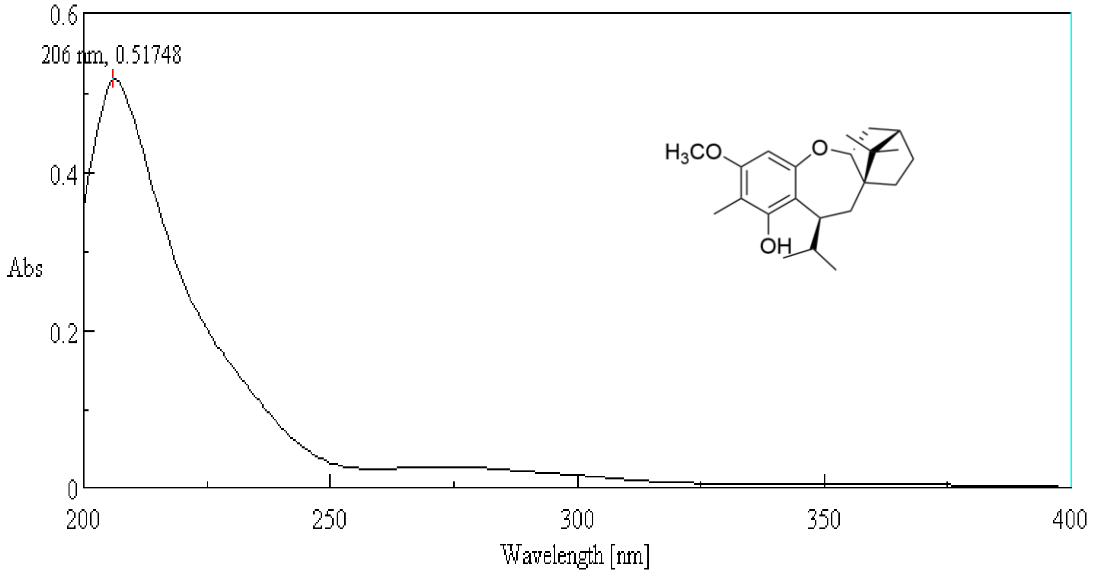

Figure S4 UV spectrum of **1** in CH_3OH

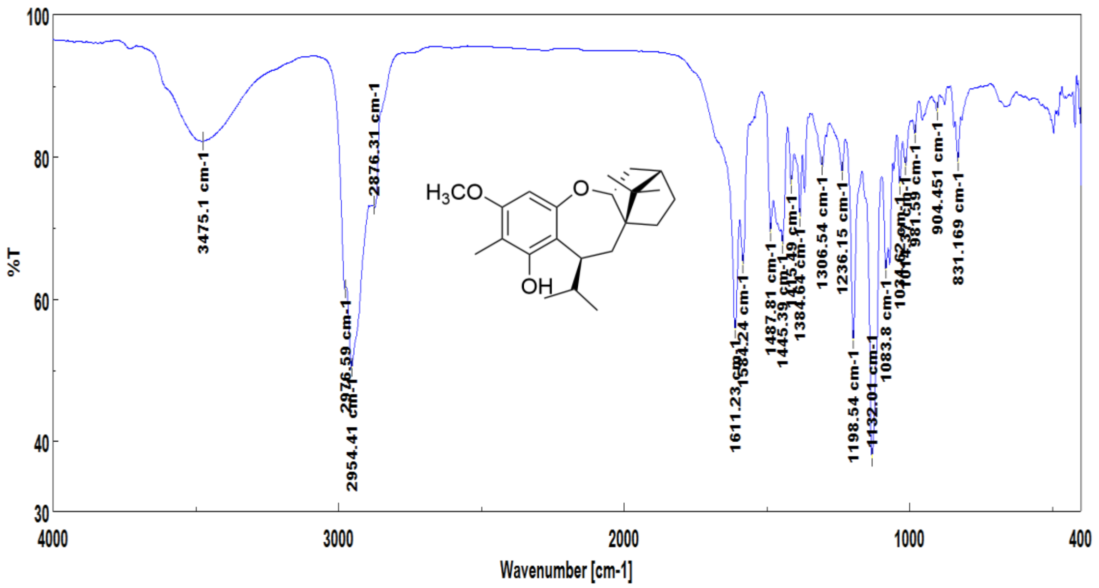

Figure S5 IR (KBr disc) spectrum of **1**

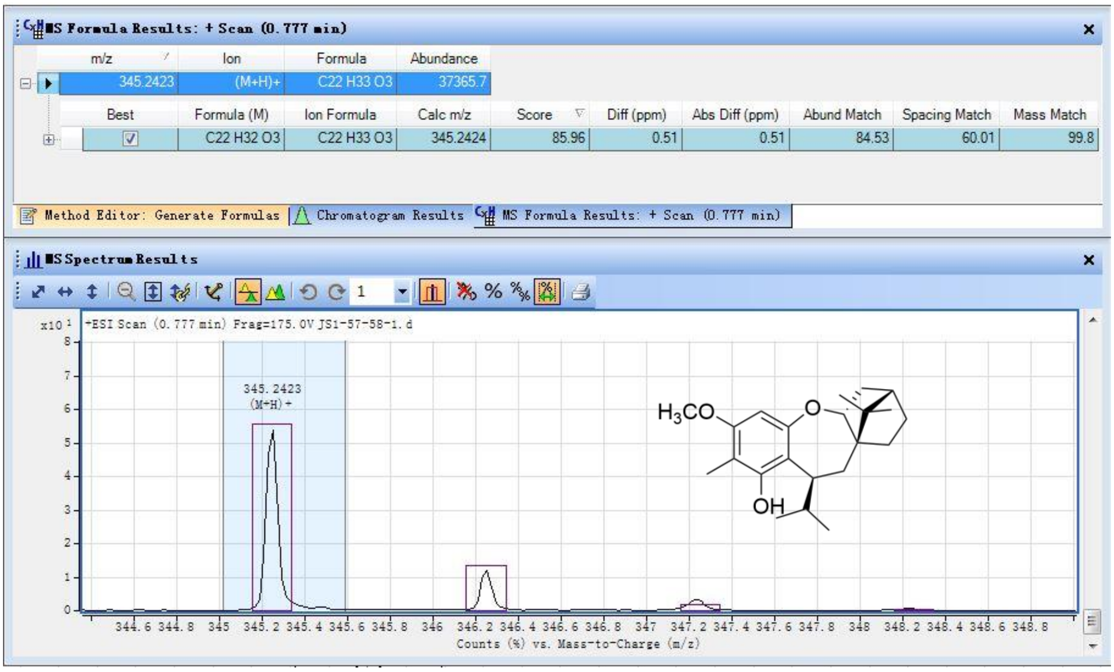

Figure S6 HRESIMS spectrum of **1** in CH_3OH

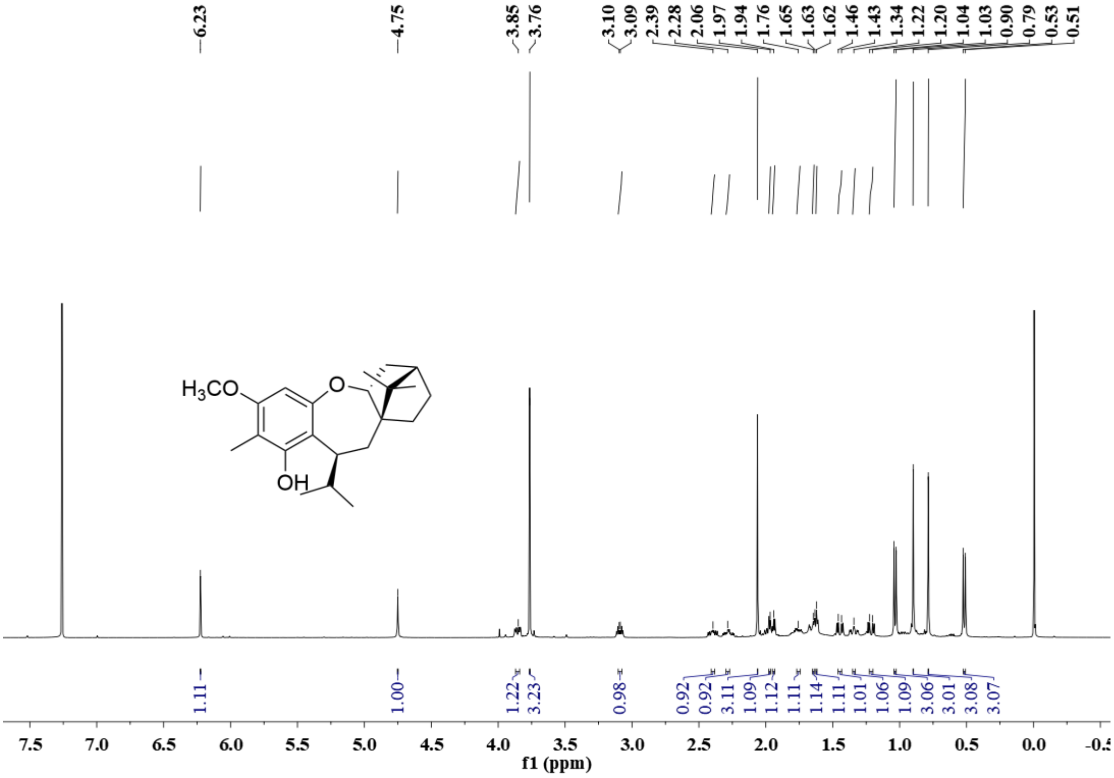

Figure S7 ^{1}H NMR spectrum of **1** in $CDCl_3$

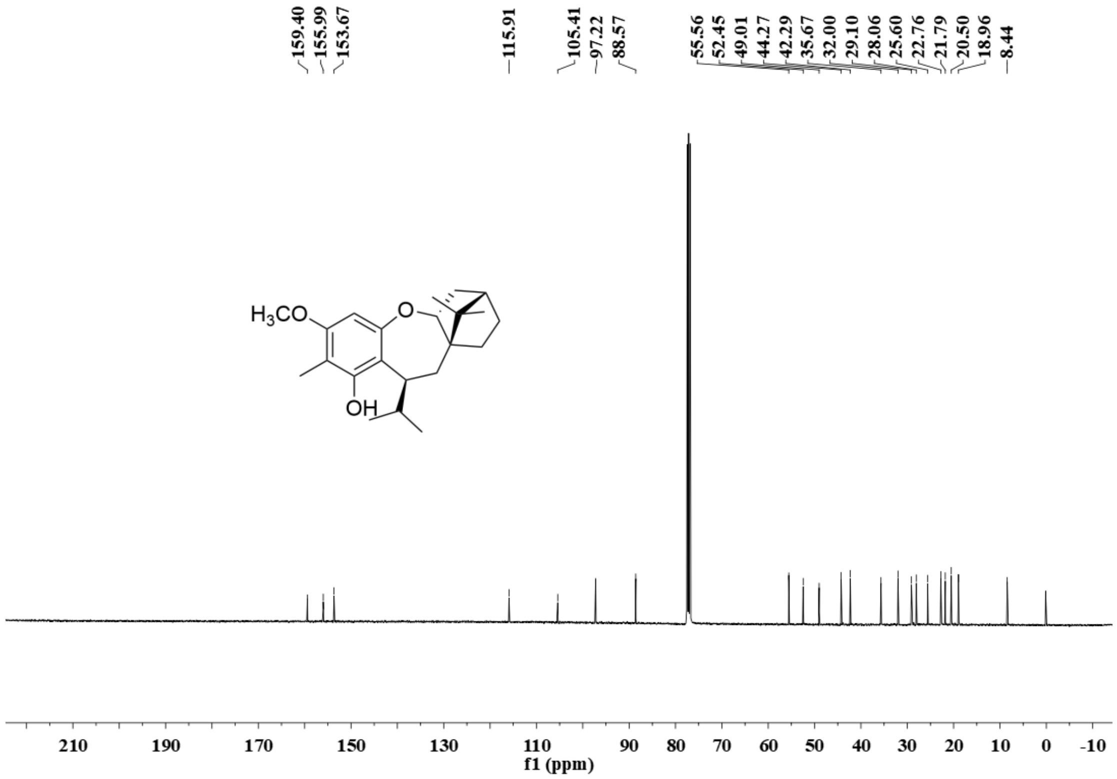

Figure S8 ^{13}C NMR spectrum of **1** in $CDCl_3$

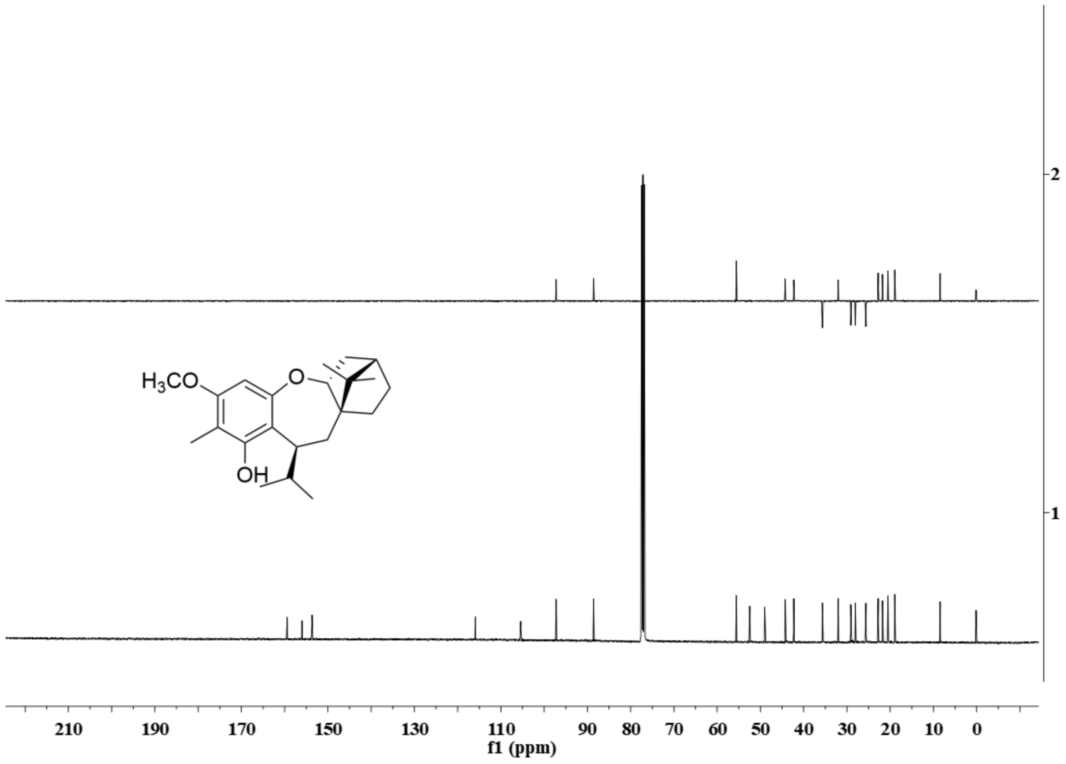

Figure S9 DEPT-135 spectrum of **1** in $CDCl_3$

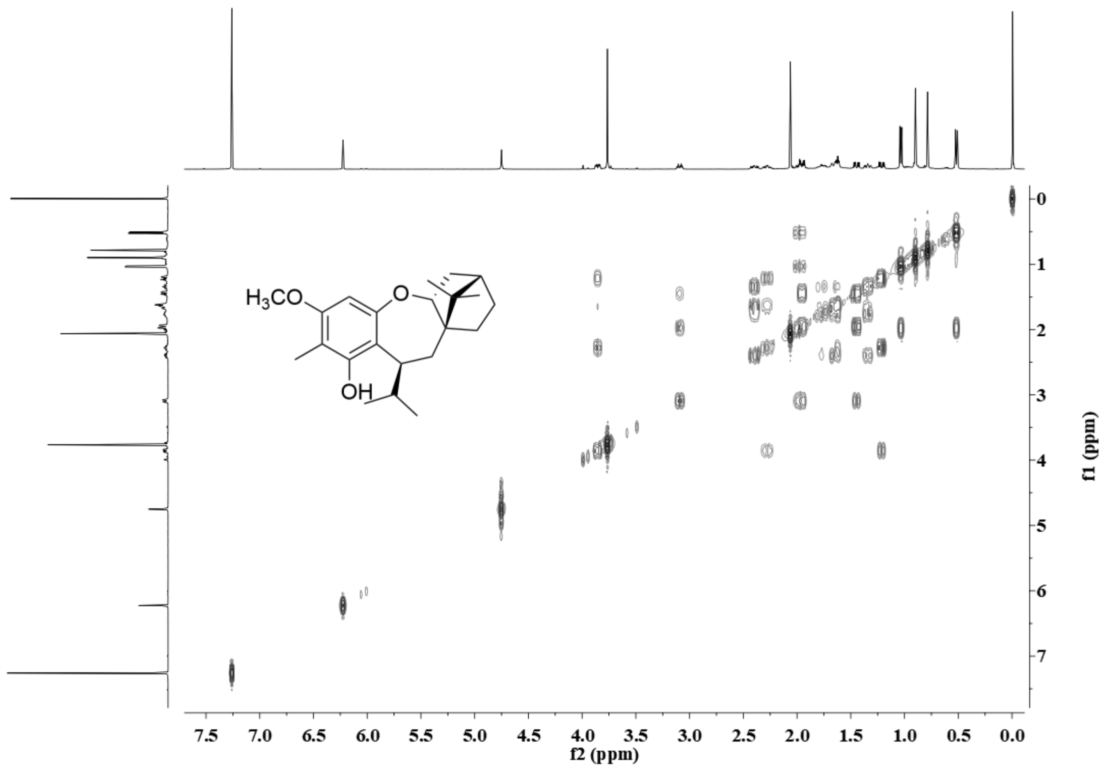

Figure S10 ^{1}H–^{1}H COSY spectrum of **1** in $CDCl_3$

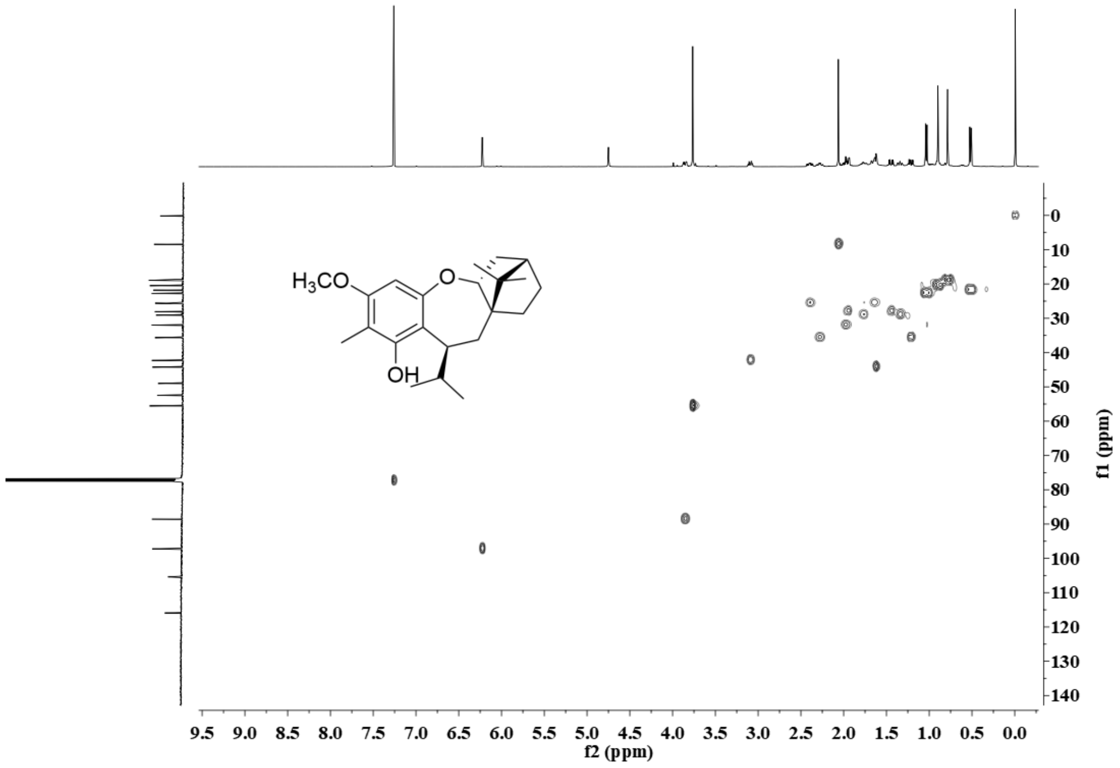

Figure S11 HSQC spectrum of **1** in $CDCl_3$

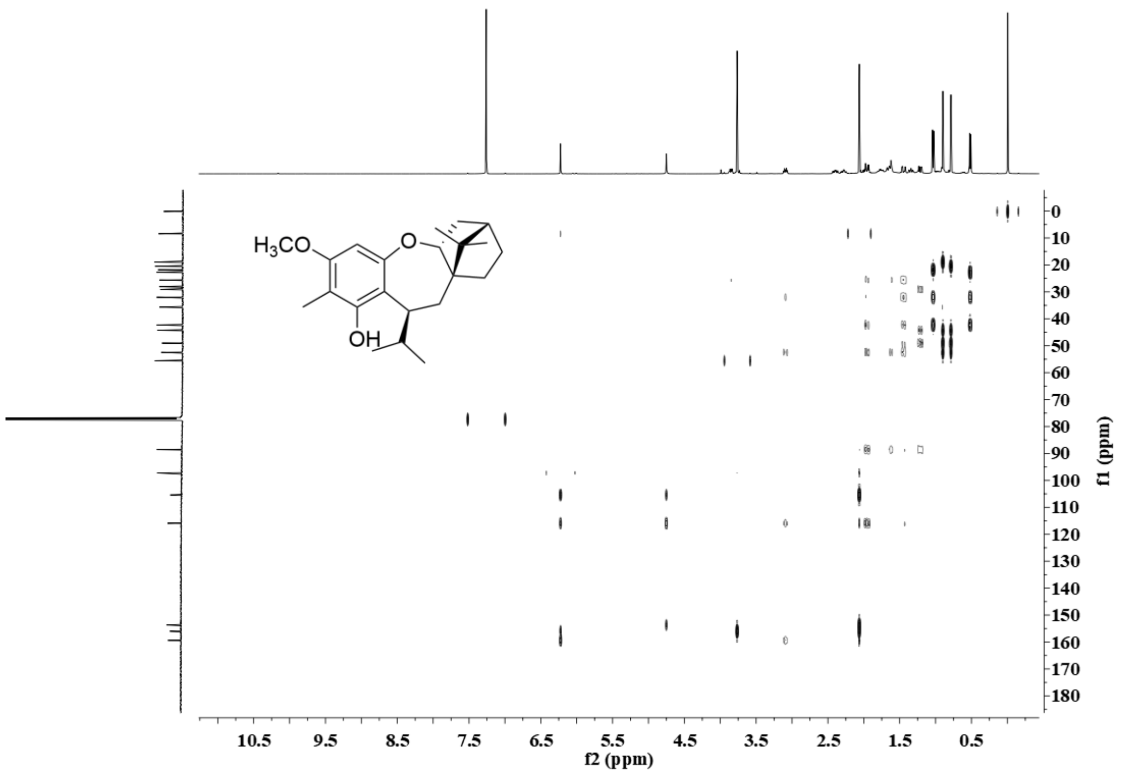

Figure S12 HMBC spectrum of **1** in $CDCl_3$

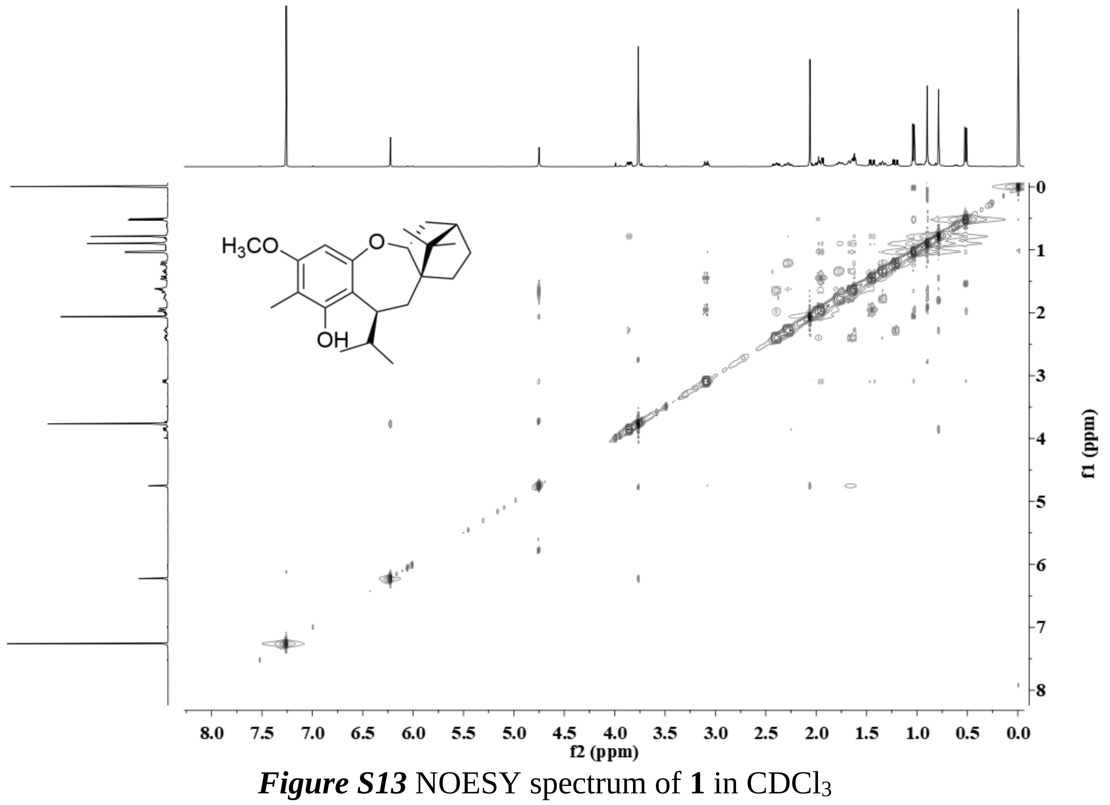

Figure S13 NOESY spectrum of **1** in $CDCl_3$

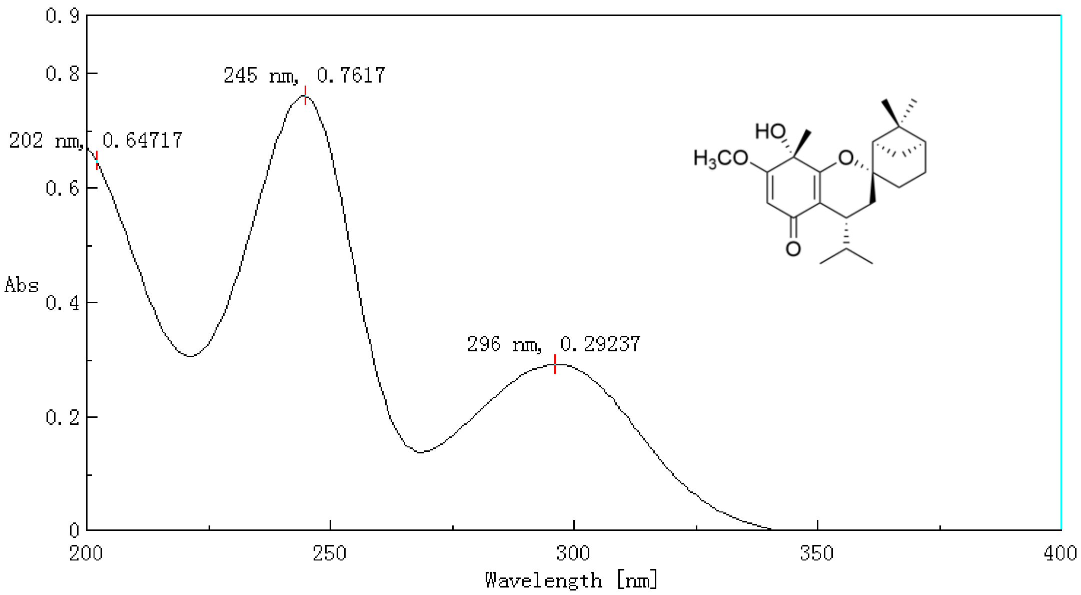

Figure S14 UV spectrum of **2** in CH_3OH

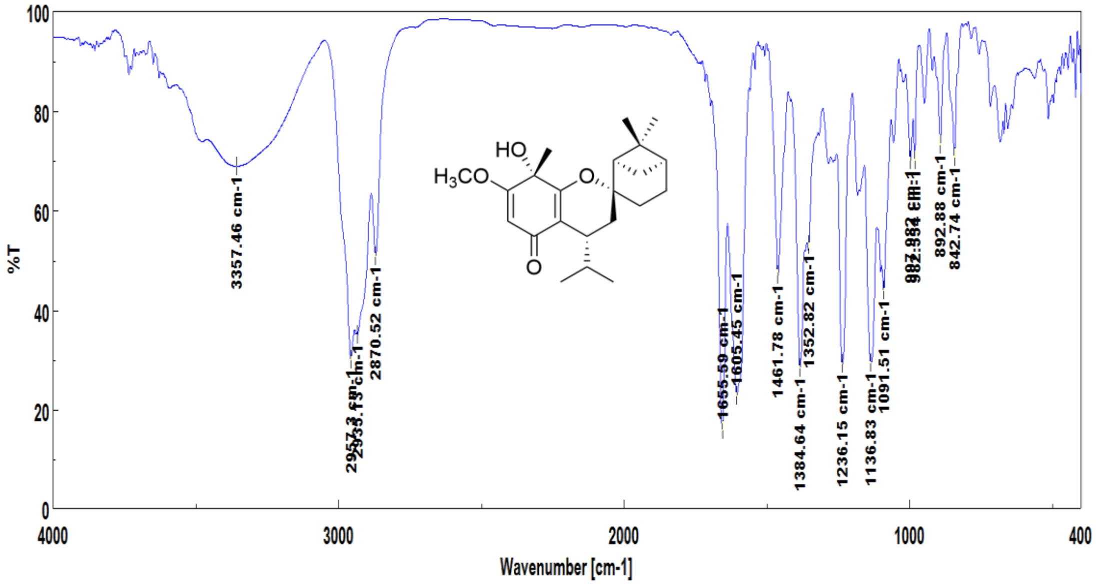

Figure S15 IR (KBr disc) spectrum of **2**

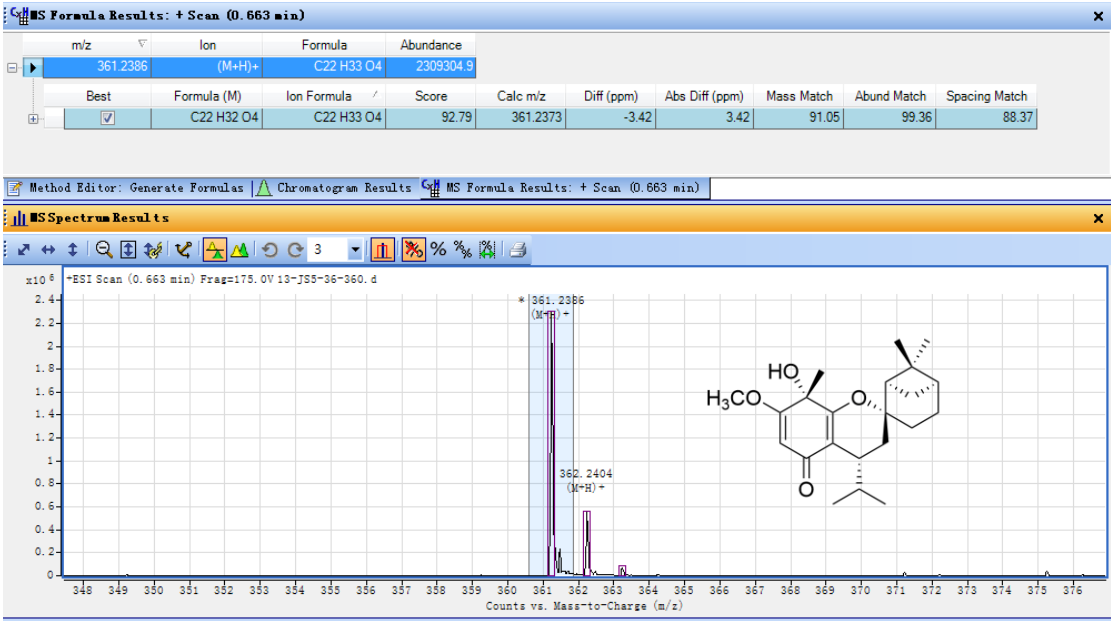

Figure S16 HRESIMS spectrum of **2** in CH_3OH

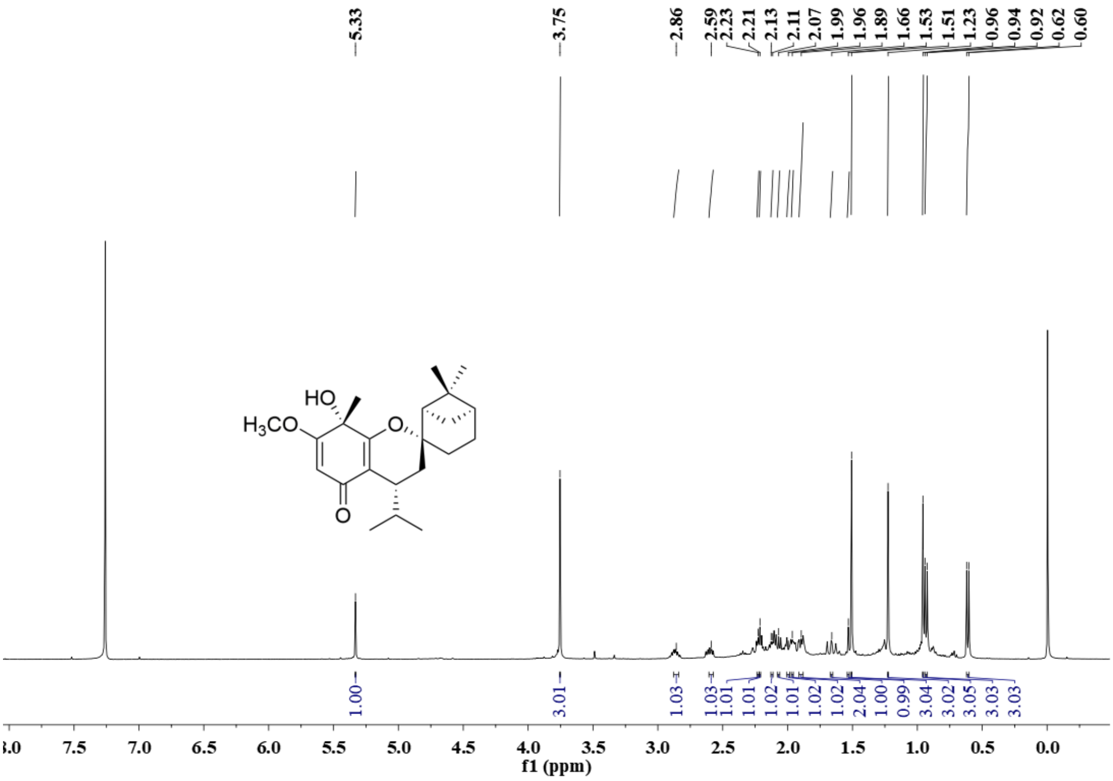

Figure S17 ^{1}H NMR spectrum of **2** in $CDCl_3$

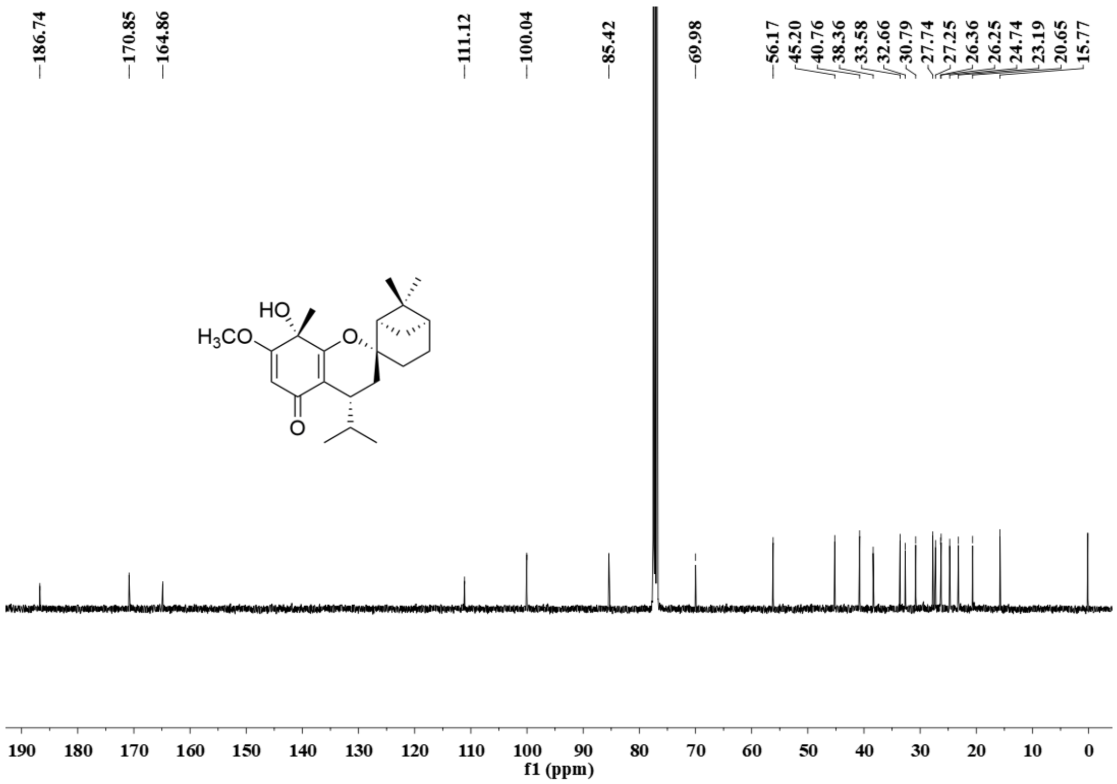

Figure S18 ^{13}C NMR spectrum of **2** in $CDCl_3$

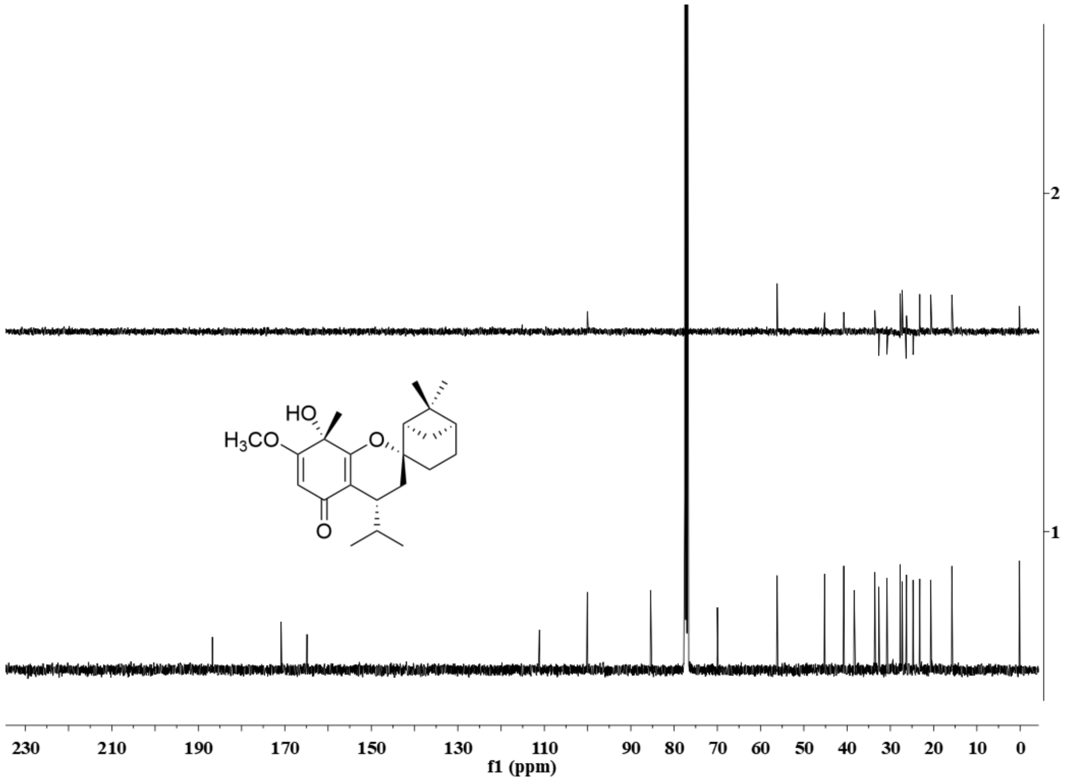

Figure S19 DEPT-135 spectrum of **2** in $CDCl_3$

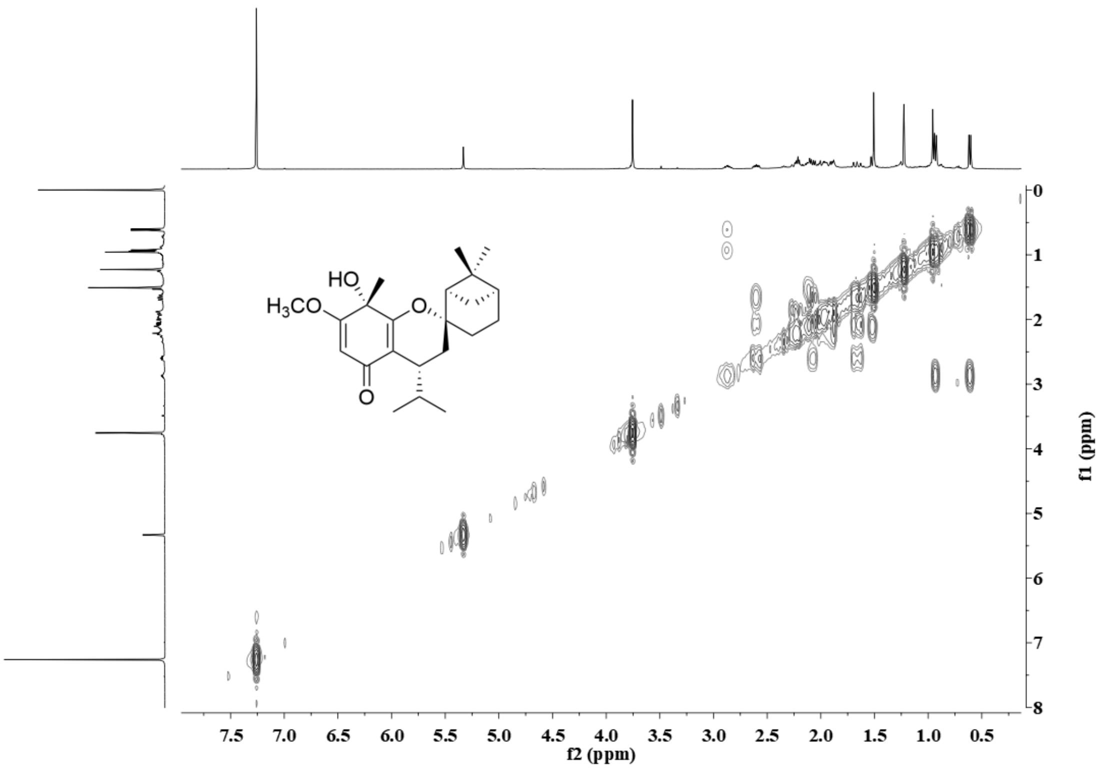

Figure S20 ^{1}H–^{1}H COSY spectrum of **2** in $CDCl_3$

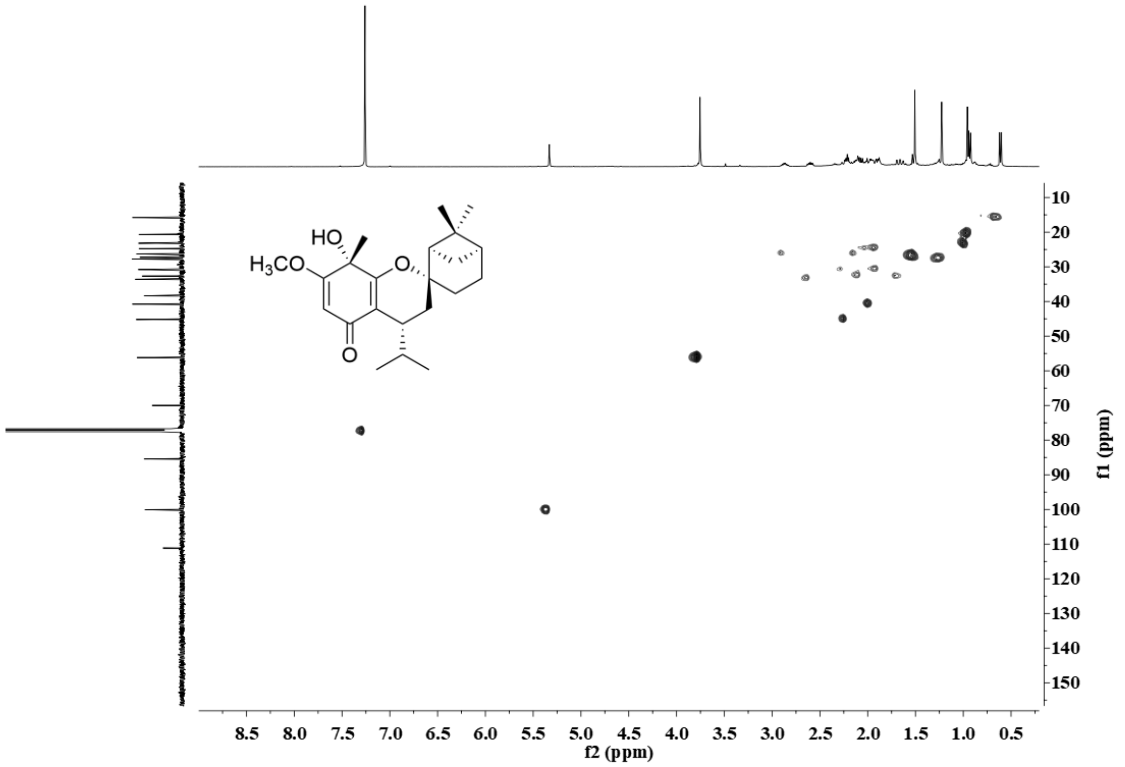

Figure S21 HSQC spectrum of **2** in $CDCl_3$

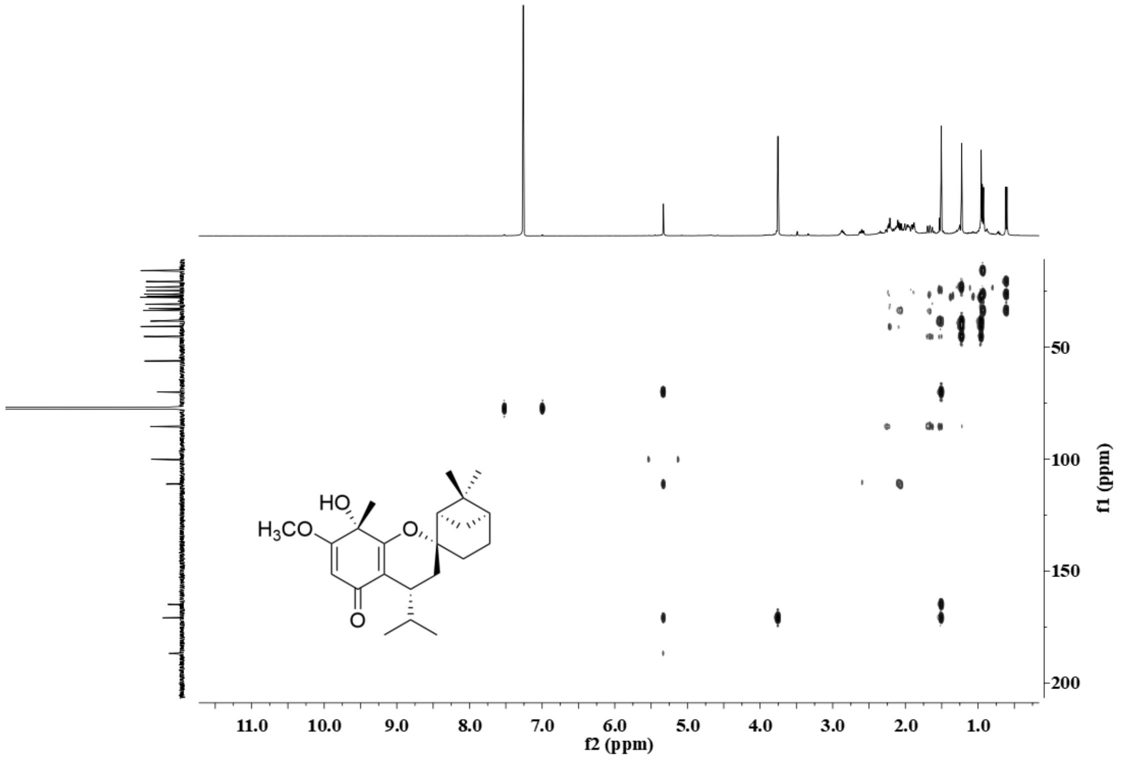

Figure S22 HMBC spectrum of **2** in $CDCl_3$

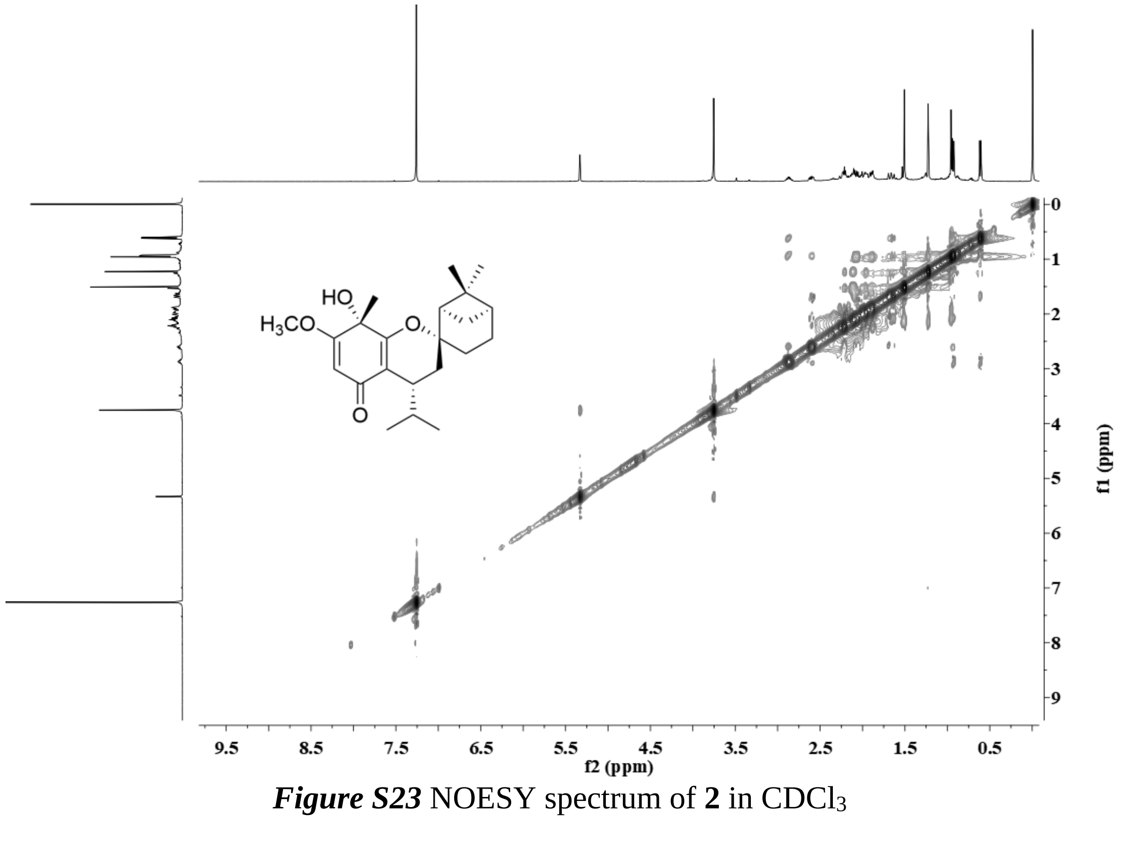

Figure S23 NOESY spectrum of **2** in $CDCl_3$

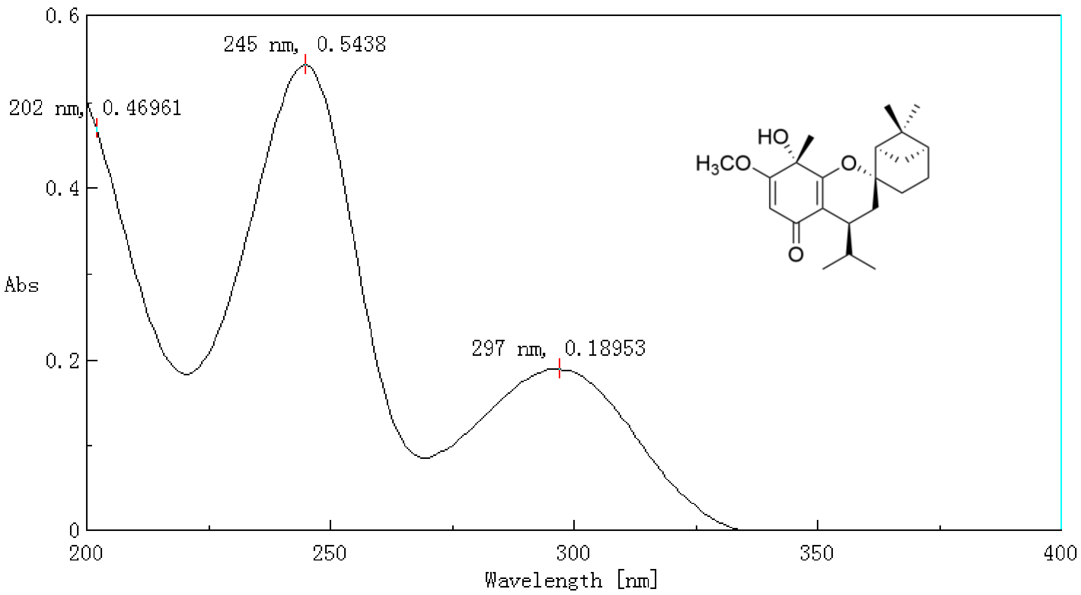

Figure S24 UV spectrum of **3** in CH_3OH

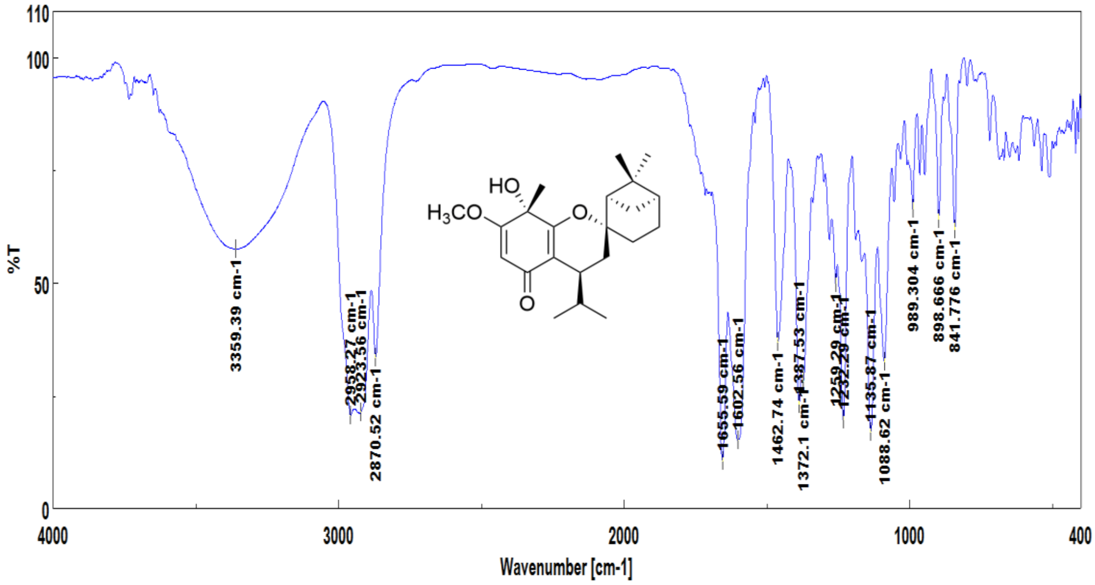

Figure S25 IR (KBr disc) spectrum of **3**

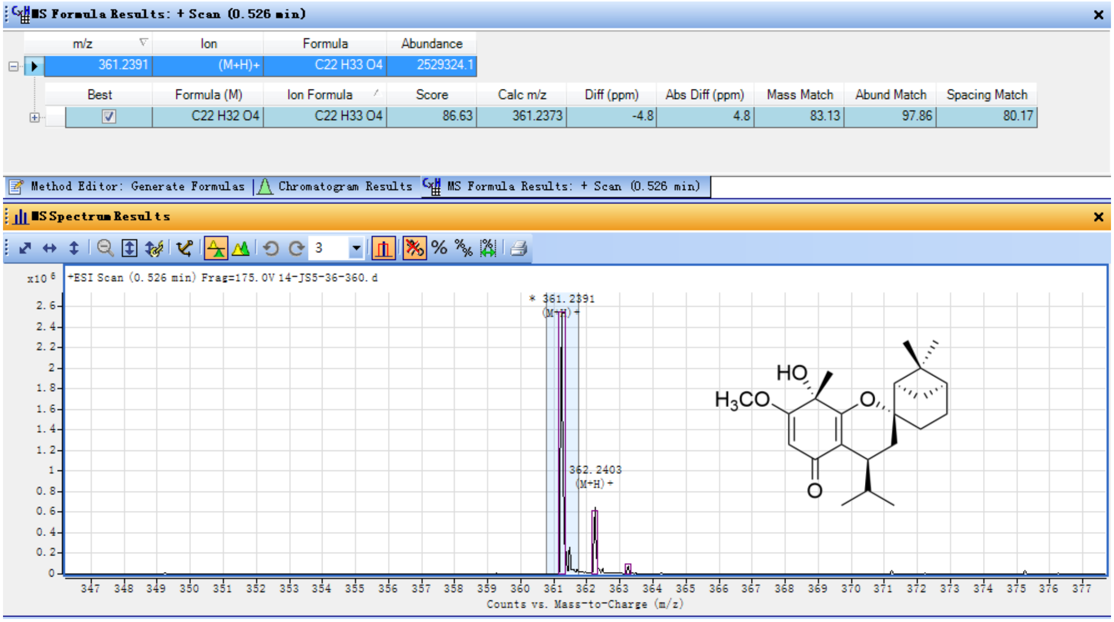

Figure S26 HRESIMS spectrum of **3** in CH_3OH

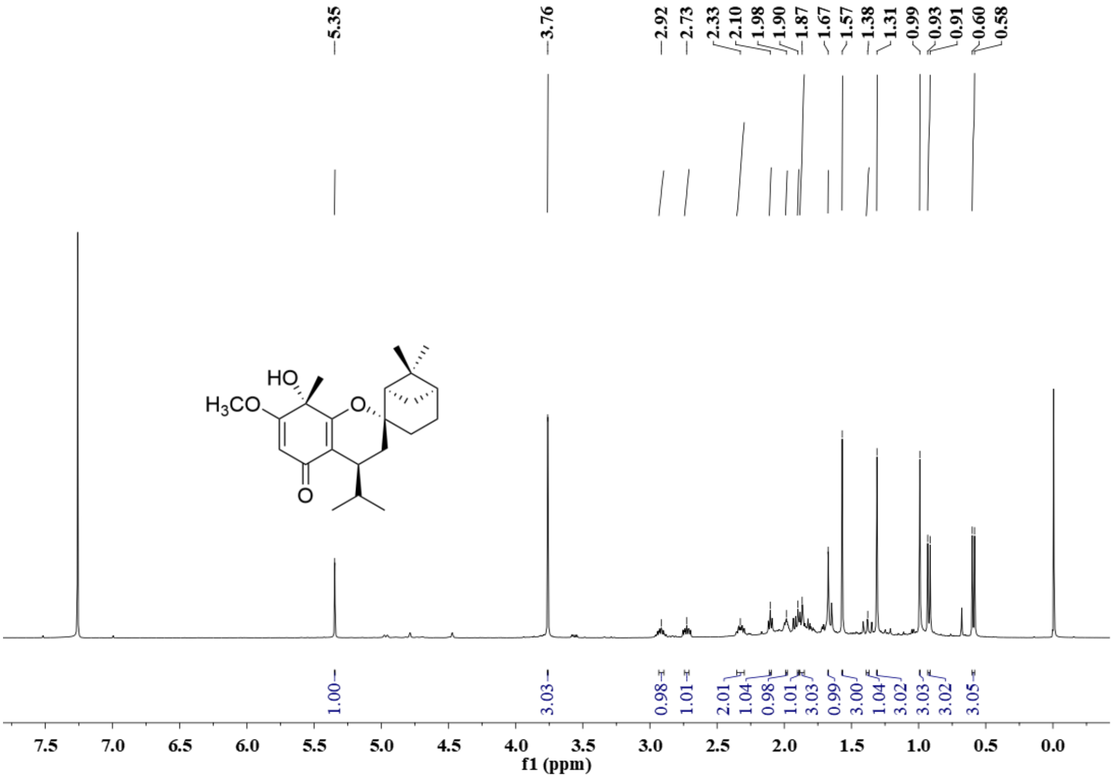

Figure S27 ^{1}H NMR spectrum of **3** in $CDCl_3$

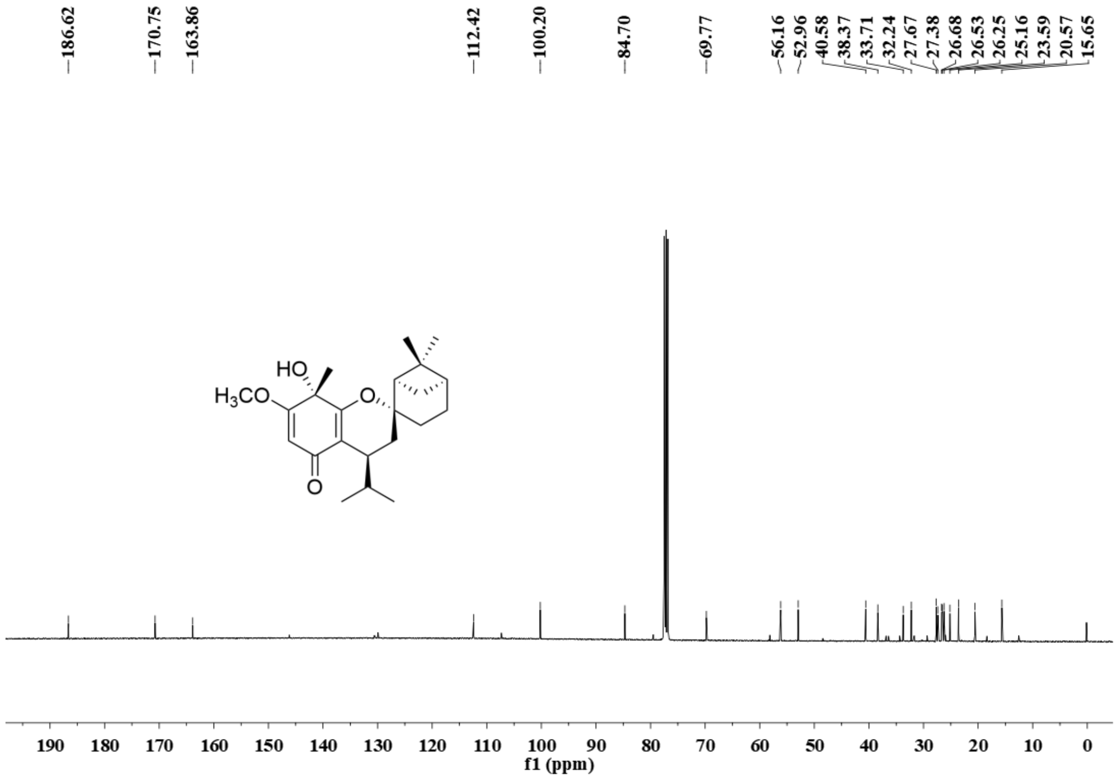

Figure S28 ^{13}C NMR spectrum of **3** in $CDCl_3$

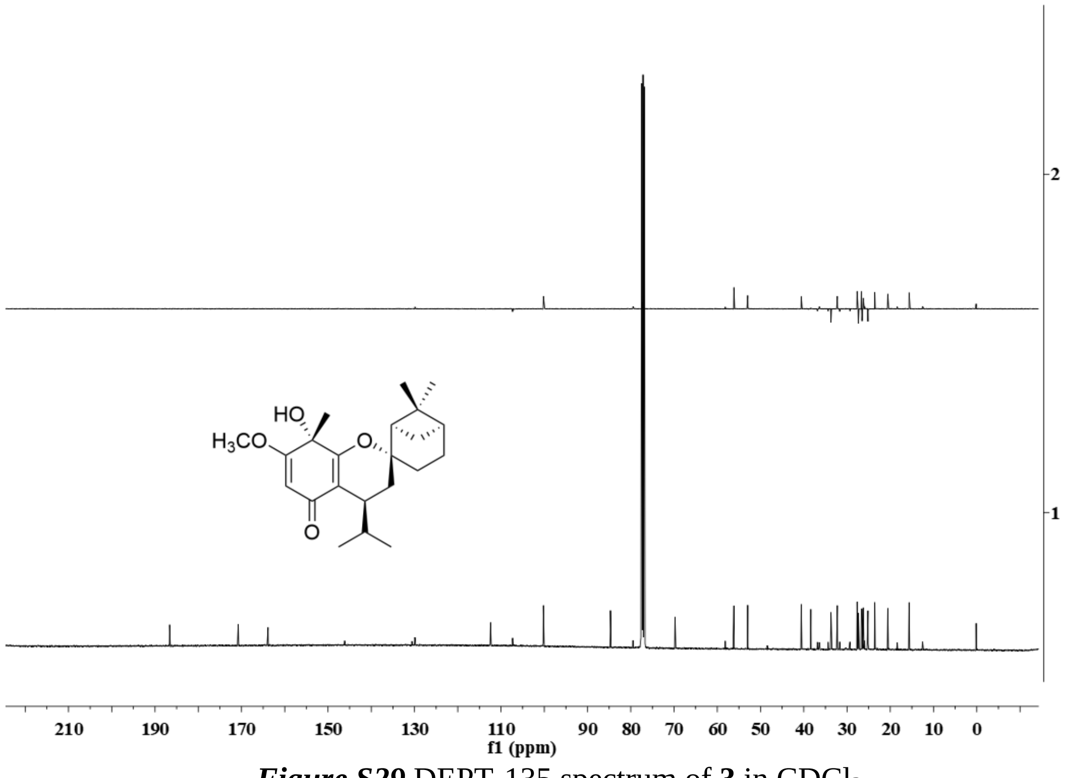

Figure S29 DEPT-135 spectrum of **3** in $CDCl_3$

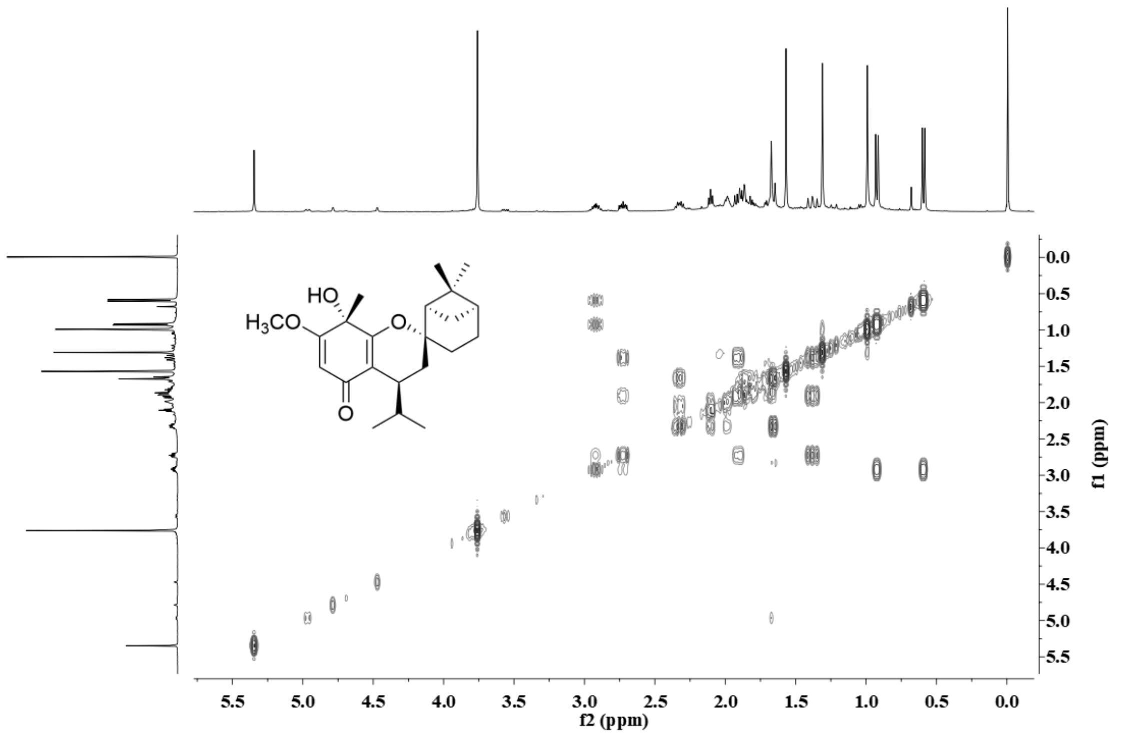

Figure S30 ^{1}H–^{1}H COSY spectrum of **3** in $CDCl_3$

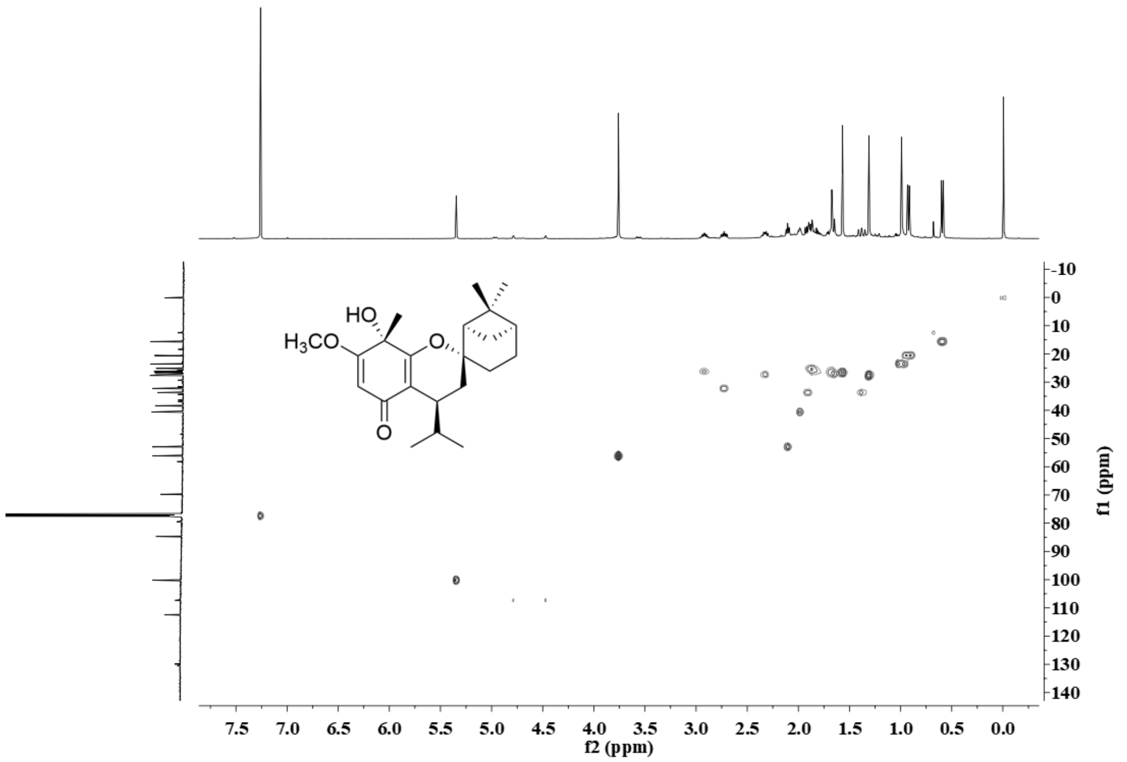

Figure S31 HSQC spectrum of **3** in $CDCl_3$

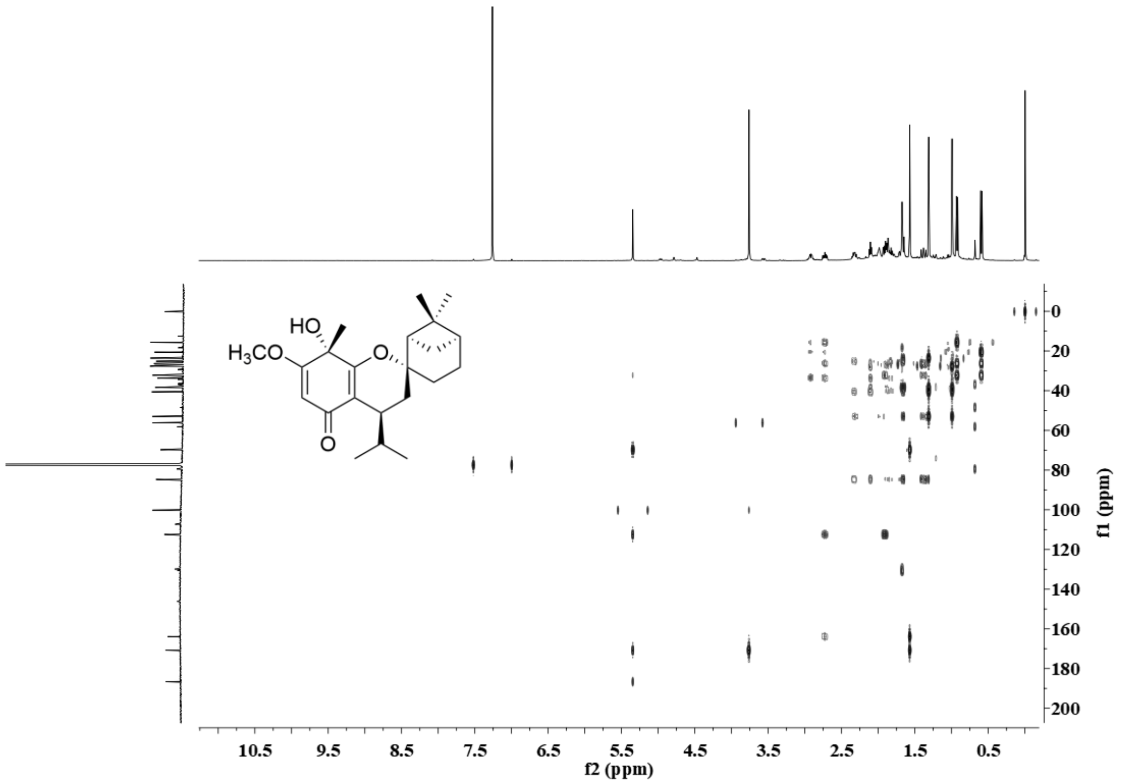

Figure S32 HMBC spectrum of **3** in $CDCl_3$

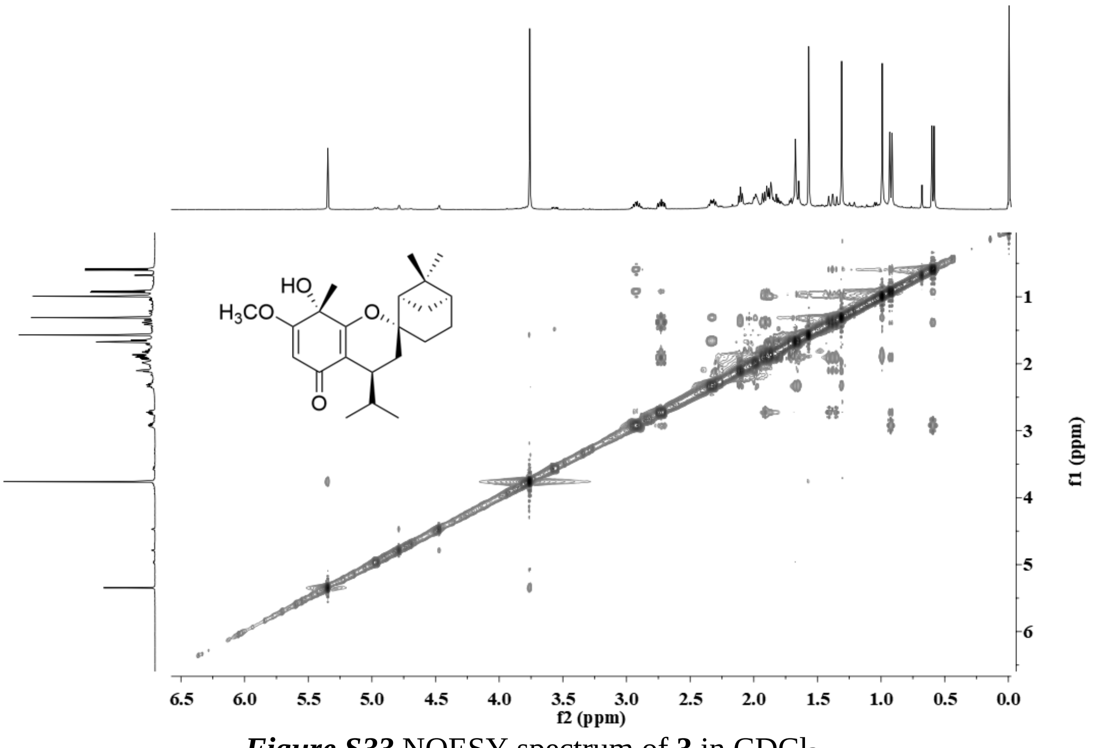

Figure S33 NOESY spectrum of **3** in $CDCl_3$

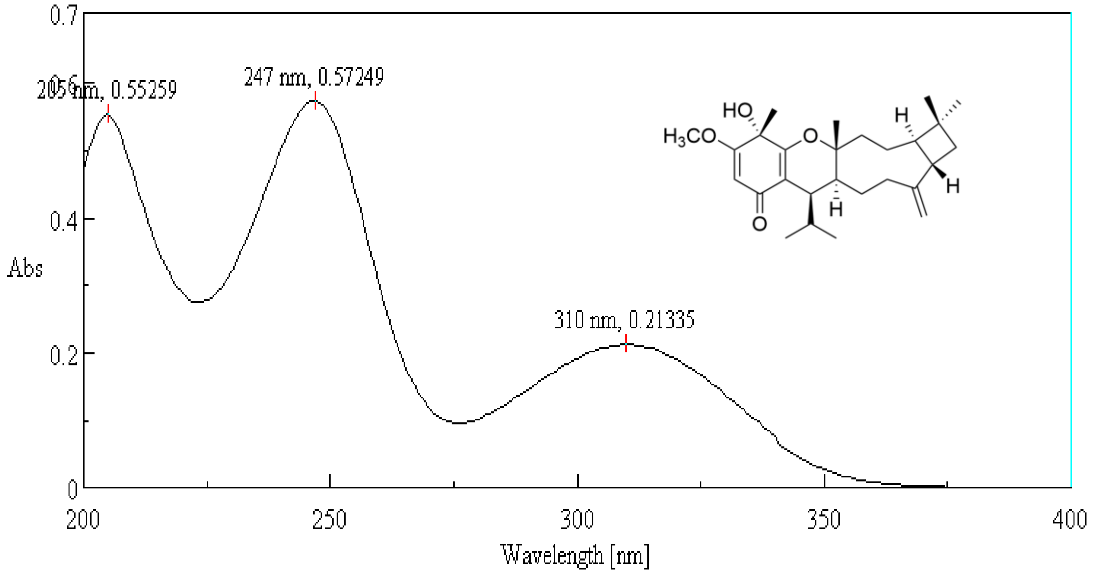

Figure S34 UV spectrum of **4** in CH_3OH

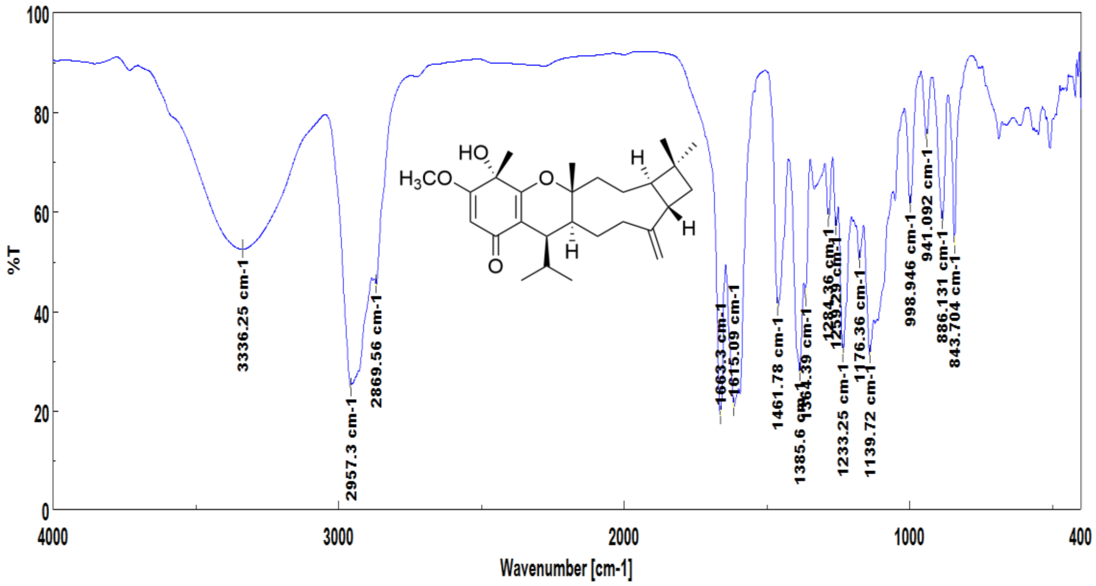

Figure S35 IR (KBr disc) spectrum of **4**

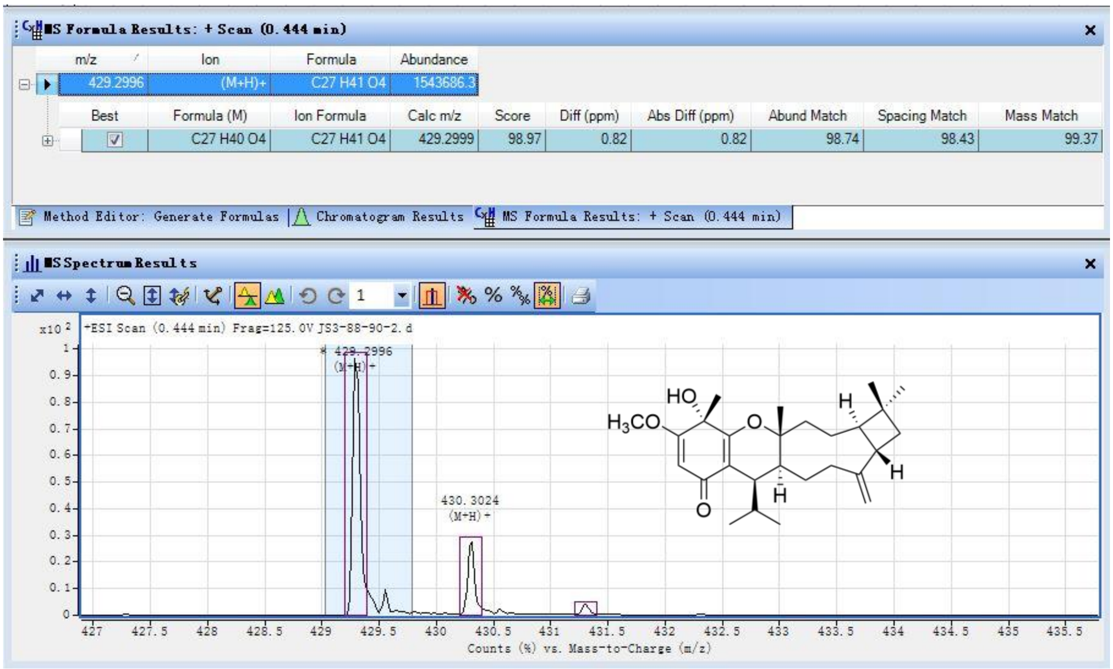

Figure S36 HRESIMS spectrum of **4** in CH_3OH

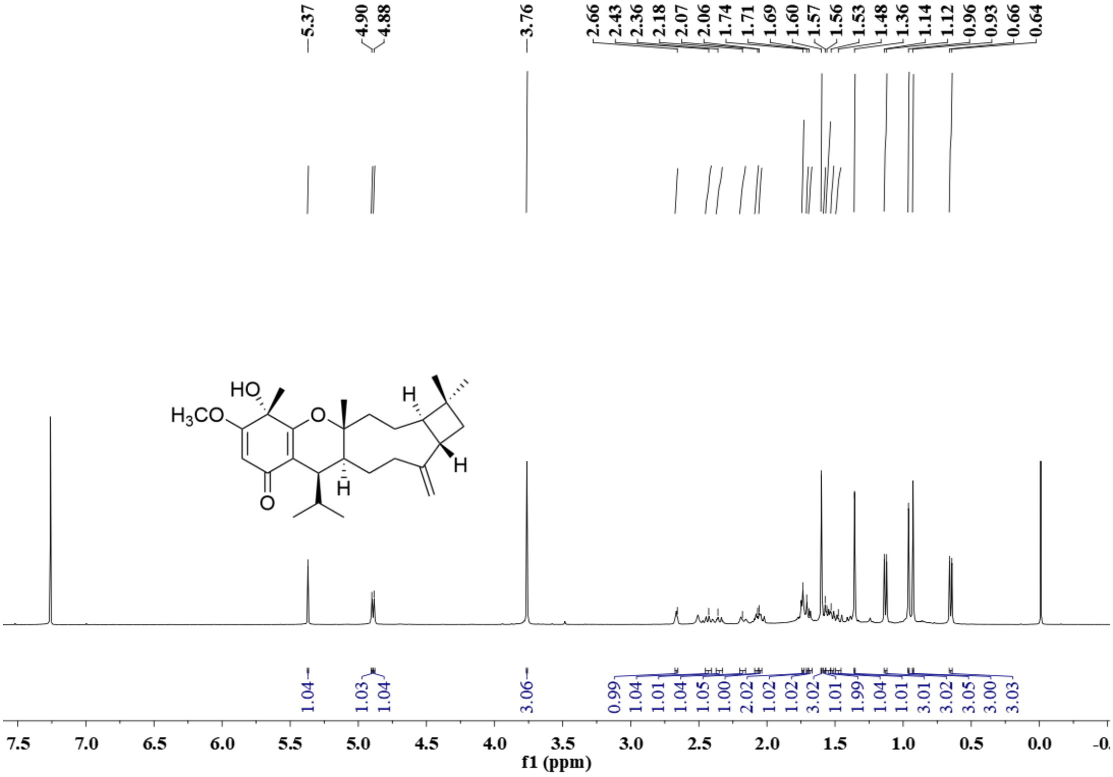

Figure S37 ^{1}H NMR spectrum of **4** in $CDCl_3$

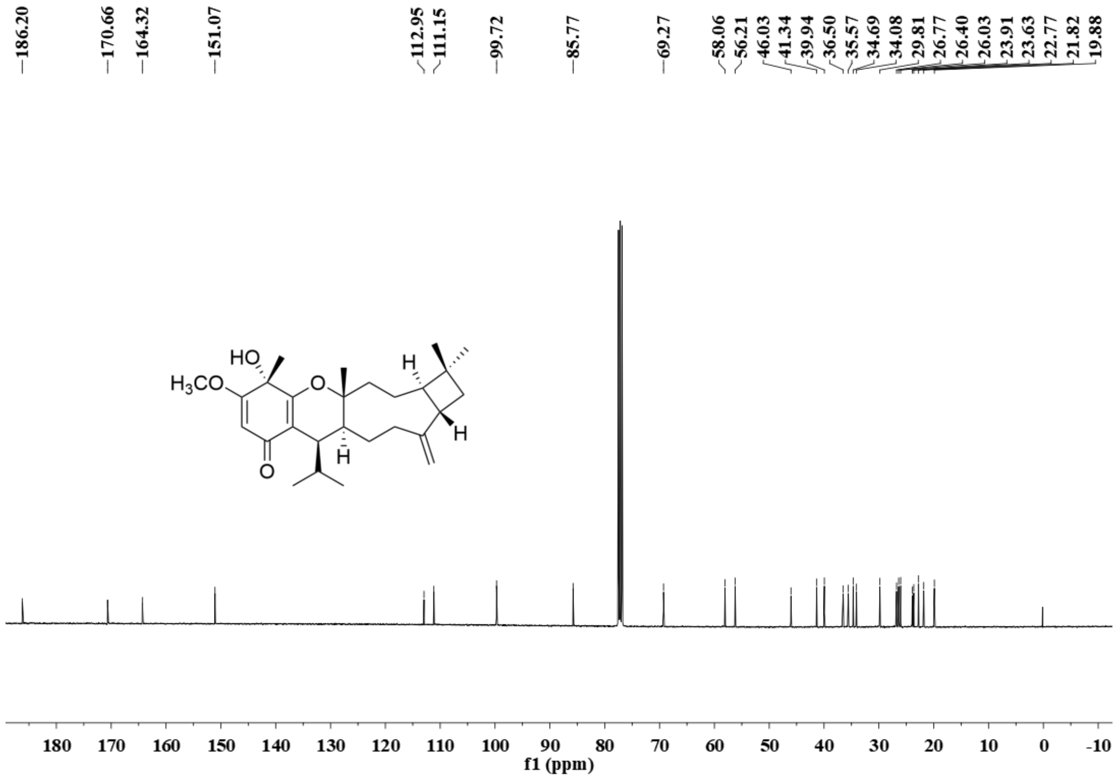

Figure S38 ^{13}C NMR spectrum of **4** in $CDCl_3$

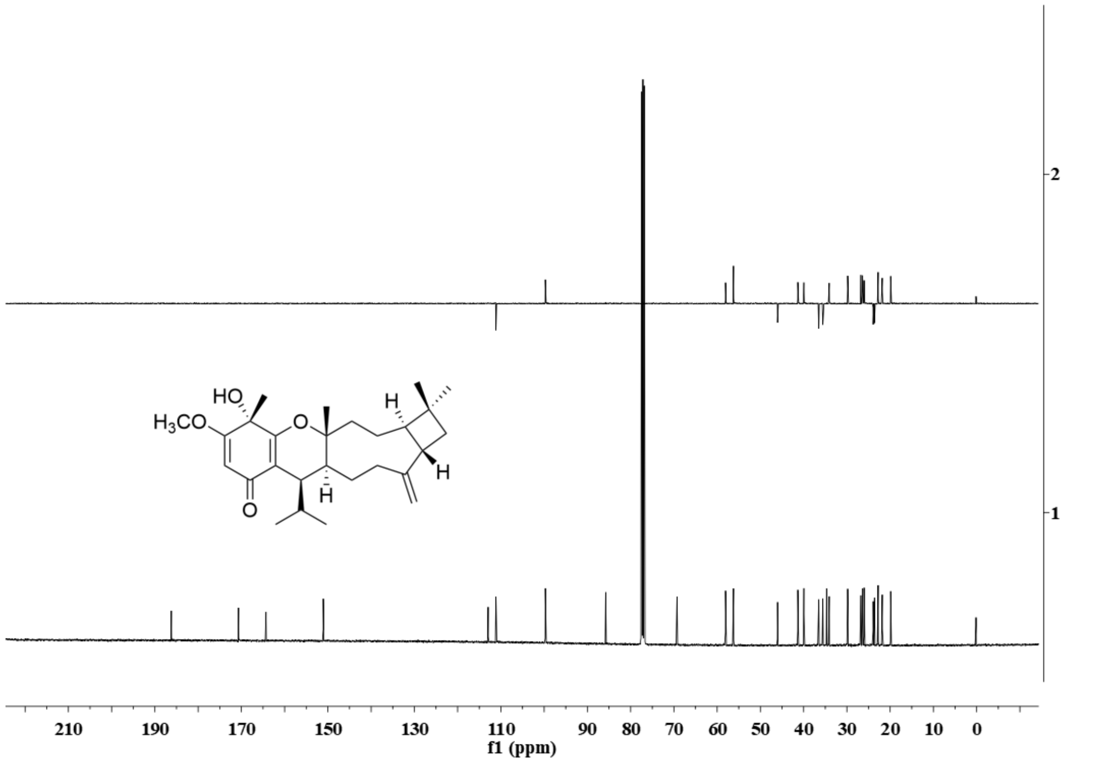

Figure S39 DEPT-135 spectrum of **4** in $CDCl_3$

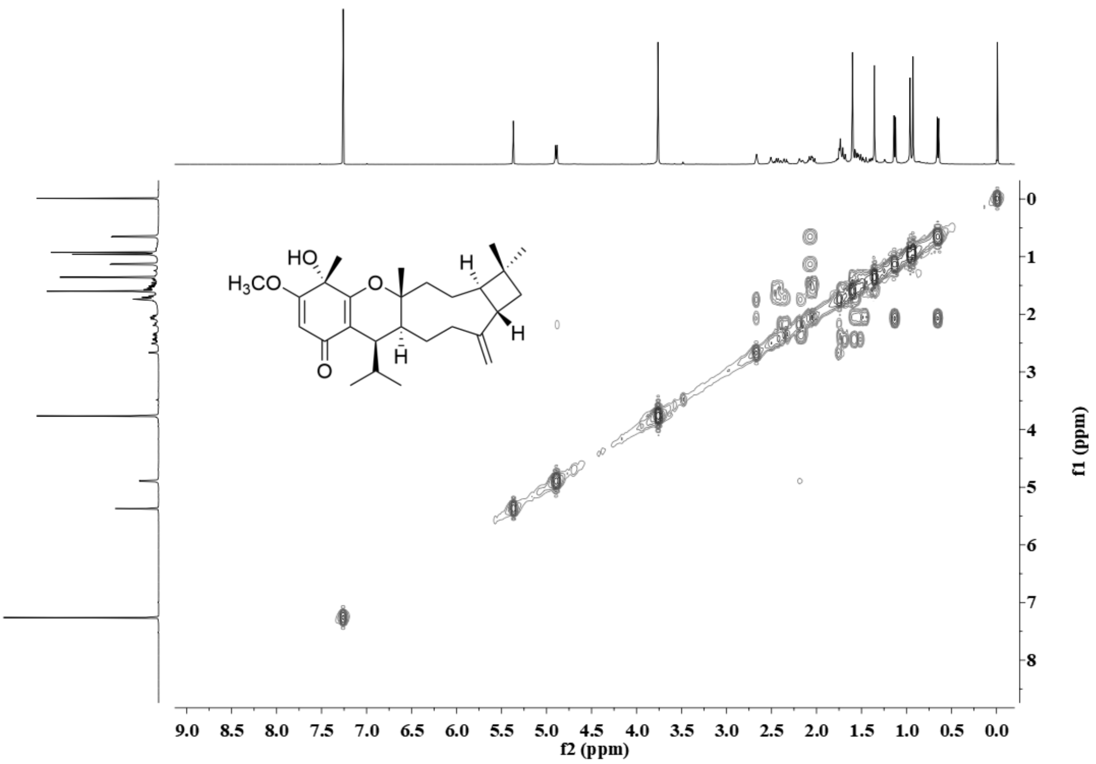

Figure S40 ^{1}H–^{1}H COSY spectrum of **4** in $CDCl_3$

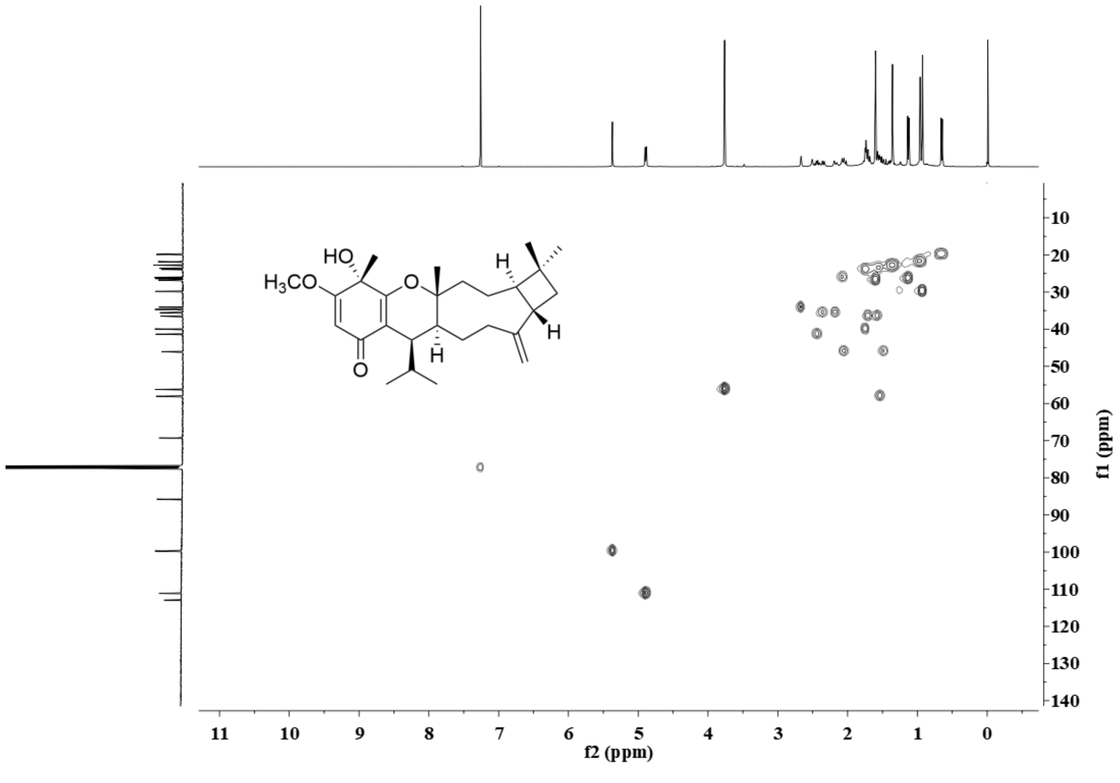

Figure S41 HSQC spectrum of **4** in $CDCl_3$

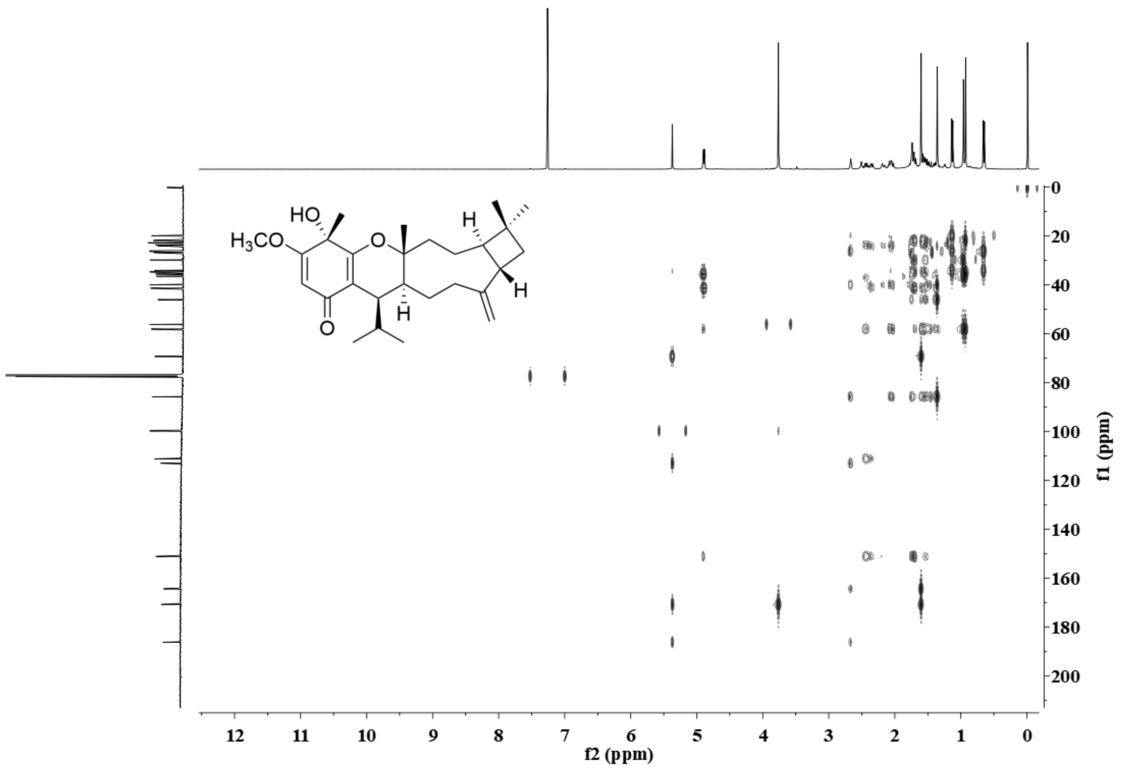

Figure S42 HMBC spectrum of **4** in $CDCl_3$

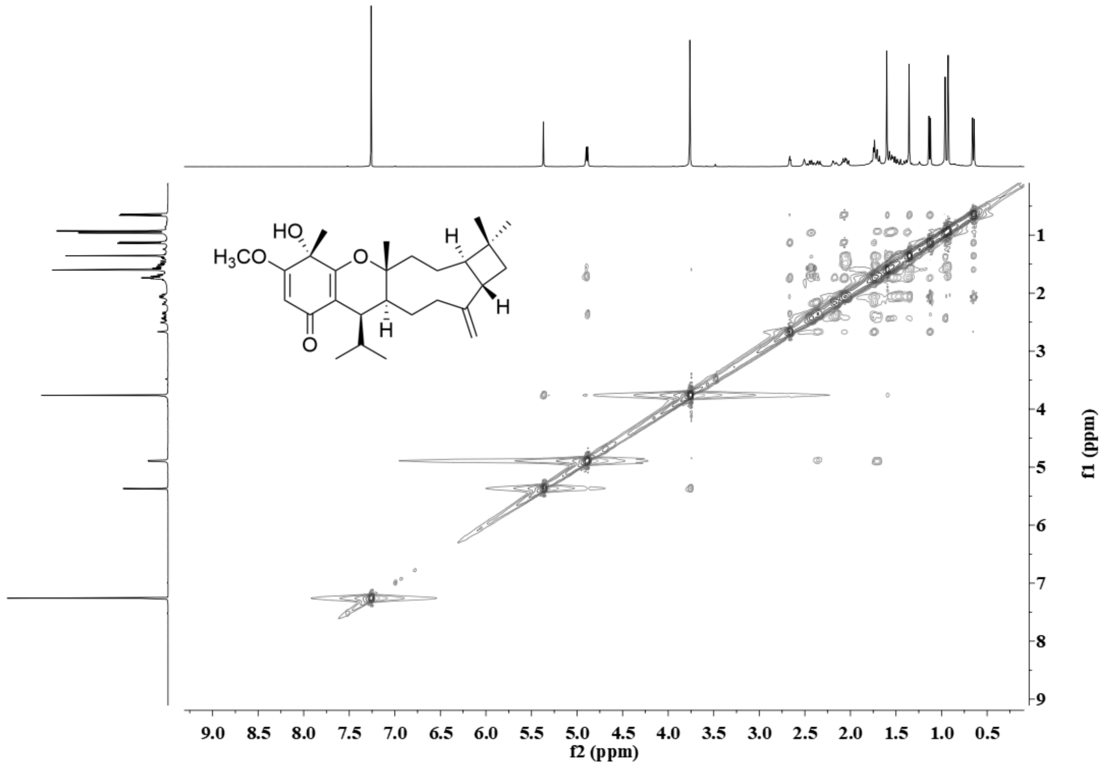

Figure S43 NOESY spectrum of **4** in $CDCl_3$
